# Effect of Nurse Home Visits vs. Usual Care on Reducing Intimate Partner Violence in Young High-Risk Pregnant Women: A Randomized Controlled Trial

**DOI:** 10.1371/journal.pone.0078185

**Published:** 2013-10-21

**Authors:** Jamila Mejdoubi, Silvia C. C. M. van den Heijkant, Frank J. M. van Leerdam, Martijn W. Heymans, Remy A. Hirasing, Alfons A. M. Crijnen

**Affiliations:** 1 EMGO+ Institute for Health and Care Research, VU University Medical Center, Department of Public and Occupational Health, Amsterdam, The Netherlands; 2 VU University Medical Center, Department of Epidemiology and Biostatistics, Amsterdam, The Netherlands; 3 de Waag, Center for Forensic Services, Amsterdam, The Netherlands; Massachusetts General Hospital, United States of America

## Abstract

**Background:**

Expectant mothers and mothers of young children are especially vulnerable to intimate partner violence (IPV). The nurse-family partnership (NFP) is a home visitation program in the United States effective for the prevention of adverse child health outcomes. Evidence regarding the effect of nurse home visiting on IPV is inconsistent. This study aims to study the effect of VoorZorg, the Dutch NFP, on IPV.

**Methods:**

A random sample of 460 eligible disadvantaged women <26 years, with no previous live births, was randomized. Women in the control group (C; n=223) received usual care; women in the intervention group (I; n=237) received usual care plus nurse home visits periodically during pregnancy and until the child’s second birthday.

**Results:**

At 32 weeks of pregnancy, women in the intervention group self-reported significantly less IPV victimization than women in the control group in: level 2 psychological aggression (C: 56% vs. I: 39%), physical assault level 1 (C: 58% vs. I: 40%) and level 2 (C: 31% vs. I: 20%), and level 1 sexual coercion (C: 16% vs. I: 8%). Furthermore, women in the intervention group reported significantly less IPV perpetration in: level 2 psychological aggression (C: 60% vs. I: 46%), level 1 physical assault (C: 65% vs. I: 52%), and level 1 injury (C: 27% vs. I: 17%). At 24 months after birth, IPV victimization was significantly lower in the intervention group for level 1 physical assault (C: 44% vs. I: 26%), and IPV perpetration was significantly lower for level 1 sexual assault (C: 18% vs. I: 3%). Multilevel analyses showed a significant improvement in IPV victimization and perpetration among women in the intervention group at 24 months after birth.

**Conclusion:**

VoorZorg, compared with the usual care, is effective in reducing IPV during pregnancy and in the two years after birth among young high-risk women.

**Trial Registration:**

Dutch Trial Register NTR854 http://www.trialregister.nl/trialreg/admin/rctview.asp?TC=854

## Background

Expectant mothers and young mothers are vulnerable to intimate partner violence (IPV)[1–3]. IPV is associated with physical injury, heart problems, gastrointestinal diseases, psychosocial problems, substance abuse, sexual risk behavior, suicide attempts, and mortality [4,5]. IPV during pregnancy increases a mother’s risk of adverse pregnancy outcomes and the likelihood that her children will develop conduct problems[6,7]. Parents involved in an aggressive relationship are more likely to abuse their child [8]. For children, both experiencing abuse and witnessing abuse are forms of child abuse. It is estimated that among young adult women, IPV is more prevalent than it is among adult women. Pregnant adolescents are approximately six times more likely to be victim of violence by a dating partner compared with their non-pregnant peers[9]. Among pregnant adolescents the prevalence of IPV ranges from 5% to 38%[10]. To protect at-risk mothers and their children from the health and developmental risks of IPV, early intervention is important, if possible, during pregnancy.

Targeted interventions designed to prevent or reduce IPV victimization and perpetration are scarce[11,12]. The Nurse-Family Partnership (NFP), developed by D. Olds et al., is a well-known nurse home visitation program that has been tested in three randomized controlled trials (RCT) with young high-risk pregnant women[13]. The trials were conducted in three distinct populations in the United States (US): Elmira (New York), Memphis (Tennessee) and Denver (Colorado)[14–17]. The NFP has proven effective for the prevention of adverse child health outcomes including child abuse. The Denver trial detected program effects on IPV at four year follow-up [18,19]. The Elmira trial also reported program effects on IPV [13]. Olds et al. showed that home visitation programs designed to prevent child abuse and neglect have limited effectiveness if the mother is currently experiencing IPV[20]. Because of the strong links between IPV and child abuse and neglect it is important to study whether nurse home visiting is effective at reducing IPV. Langhinrichsen-Rohling et al. conducted a preliminary test of an IPV prevention program among a small group of high-risk inner-city pregnant adolescent girls in which they found an effect on IPV perpetration and victimization[21]. 

In the Netherlands, the NFP was translated into the Dutch language and adapted to be integrated into the Dutch health care system. Although the adapted program, VoorZorg, is the first evaluation of the NFP outside the US, other adaptations of the program are currently being evaluated in England, Canada and Australia. VoorZorg consists of 40-60 structured home visits with young pregnant women by well-trained nurses, from pregnancy until the child is two years of age. Primary outcome measures of the Dutch RCT are smoking cessation, birth outcomes (birth weight and pregnancy duration), breast feeding, child development, IPV and child abuse [22]. The objective of the current study is to assess the effect of VoorZorg on addressing self-reported IPV victimization and perpetration among young, low-educated pregnant women and mothers compared with young mothers receiving the usual care in the Netherlands.

## Methods

The protocol for this trial and supporting CONSORT checklist are available as supporting information; see Checklist S1 and Protocol S1. Prior to this study, the NFP was translated into Dutch and culturally adapted to accommodate the needs of pregnant women in the Netherlands and to be integrated into the Dutch child health care system[22]. The most important adaptations were placing more emphasis on home delivery, instructing women to stop smoking during pregnancy, offering more information about breastfeeding and emphasizing the advantages of breastfeeding, adjusting program practices to avoid overlapping duties with midwifes or youth health care professionals, organization of pregnancy classes, ultrasounds and other educational opportunities; these files are available as supporting information; see Adjustments in the Dutch Version of Pregnancy Guidelines S1 and Adjustments in the Dutch Version of Infancy Guidelines S1 [23,24]. The intervention and implementation were tested in a pilot study. 

### Ethics Statement

This study was approved by the Medical Ethical Committee of the VU University Medical Center (VU MC). Women who declined to participate were not disadvantaged in any way by refusing to participate in this study. They received the usual standard of care. 

The informed consent procedure was conducted by a specialized VoorZorg nurse during the selection process, which is described in the next paragraph. The VoorZorg nurse informed the pregnant woman about the content of the VoorZorg program and the RCT, and explained the 50% chance to be assigned to the control group. If the woman agreed to participate, a written informed consent was signed after explaining the aim of signing this form. These steps were all written in a protocol designed for VoorZorg nurses. All participants signed forms acknowledging informed consent.

### Participants and setting

From 2007 to 2009, 460 participants were recruited for the RCT based on a sample size calculation. A two-stage selection procedure was performed (Unpublished data). During the first stage, midwives, general practitioners, gynecologists, and others actively recruited women in 20 municipalities in the Netherlands. Inclusion criteria for the first stage were: (1) maximum age of 25 years, (2) low educational level (pre-vocational secondary education), (3) maximum 28 weeks of gestation, (4) no previous live birth and (5) some understanding of the Dutch language. These women were routed to the second stage, in which VoorZorg nurses interviewed women to ensure they had at least one additional risk factor (being single, a history or present situation of domestic violence, psychosocial symptoms, unwanted pregnancy, financial problems, housing difficulties, no employment and/or education, alcohol and/or drug use). When a potential participant did not meet all of the inclusion criteria for the first stage, but had multiple risk factors, the VoorZorg nurse presented the case to an independent expert committee, which decided on inclusion or exclusion. The number (%) of participants recruited in this manner was 77 (16,7%).

All eligible women were randomized into the control or intervention group after stratification by region and ethnicity (Dutch, Surinamese/Antillean, Turkish, Moroccan, Cape Verdean or other). Ethnicity classification was performed by the VoorZorg nurse based on participants’ self-reports. A participant was classified as a certain ethnicity if at least one of her biological parents was born in a particular country. Finally, 223 women were assigned to the control group and 237 women to the intervention group. 

### Intervention

Women in the control group received the usual care [22]. The usual care consists of maternal health care during pregnancy offered by a midwife or obstetrician to gain optimal pregnancy outcomes. The midwife or obstetrician offers health education, performs physical examinations and monitors the development of the fetus. After birth, a maternity care helper visits the mother at home to take care of the mother, the newborn and the household, and advises the mother about taking care of her baby. Furthermore, every newborn is registered in a child health care organization (ambulatory well-baby clinic) to monitor the health and development of the child and to support parents in their new role. In total, nine to eleven check-ups are performed until the child’s second birthday. Families with special needs can receive support from (child) welfare organizations and mental health services established in different regions in the Netherlands. 

 Women in the intervention group were offered approximately 10 nurse home visits during pregnancy, 20 during the first year and 20 during the second life of the child’s life by trained and experienced VoorZorg nurses, in addition to the usual care. Text messaging, telephone and social media were also used to contact the mothers. Home visits are well-structured and described in manuals; each of six domains (health status of the mother, child's health and safety, personal development of the mother, the mother as a role model, relation of the mother with her partner, family and friends, and use of institutions) were addressed during each visit. The participant’s partner and/or father of the baby was included during each home visit, if possible. 

Ultimate goals of this structured nurse home visitation program are: to improve the outcomes of pregnancy by improving a mother’s health during pregnancy, to improve the child’s health and development by helping parents provide more competent care to their children, and to improve the mother’s own personal development. 

Within the context of these well-structured visits, several elements are considered to address IPV. The VoorZorg nurses attempted to mitigate risk factors for IPV by reducing stress, by trying to make women financially independent, or by providing housing assistance. Nurses helped women (and their partners) during home visits to be aware of IPV, to identify abusive relationships by use of the Power and Control Wheel and to make them aware of the consequences of abuse for the child [25]. The Power and Control Wheel demonstrates the different types of abuse that perpetrators use to control their victims. For safety reasons, this tool was discussed with the mother alone. Moreover, VoorZorg nurses supported women and their partners with strategies for emotional regulation and communication. The nurses also helped both partners to make safer decisions for the sake of themselves and their child, such as preventing arguments from escalating to a physical fight by teaching them how to address these situations, and by teaching them how to negotiate and to listen to each other. In families where IPV was present, these topics were repeated at each home visit. These elements match largely with the essential elements for effective programs on IPV for high-risk adolescent pregnant girls as identified by Langhinrichsen-Rohling and Turner (2012) [21]. 

 VoorZorg nurses strive to establish a trusting relationship with mothers-to-be at a sensitive time in their development. Because it takes time to establish such a relationship, risk factors for IPV and child safety were addressed over a prolonged period of time during pregnancy and first years of life. 

### Measurements

The RCT measured the following outcomes[22]:

•Maternal cigarette smoking at 16-28 weeks and 32 weeks of pregnancy and two months after birth as well as maternal smoking near the child;
•Adverse pregnancy outcomes, birth weight and gestational age;
•Child development at six months, 18 months and 24 months of age, measured with, among others, the Home Observation for Measurement of the Environment, and the Child Behavior Checklist [26] [27];
•Child abuse reports;
•Intimate Partner Violence.

This manuscript specifically addresses self-reported IPV. 

The primary outcome measure was self-reported psychological, physical or sexual violence, and injury towards the participant (victim) as well as towards her partner (perpetrator). Secondary outcomes were a summation of forms of violence, and both experiencing and perpetrating IPV. All outcomes were measured with the revised Conflict Tactics Scale. Psychometric properties are described by Straus et al. [28]

All women were interviewed three times at their home, at 16-28 and 32 weeks of pregnancy and 24 months after birth, by trained female interviewers. To prevent socially desirable answers and for safety reasons, the interviewers conducted the interviews in private. 

**Figure 1:**
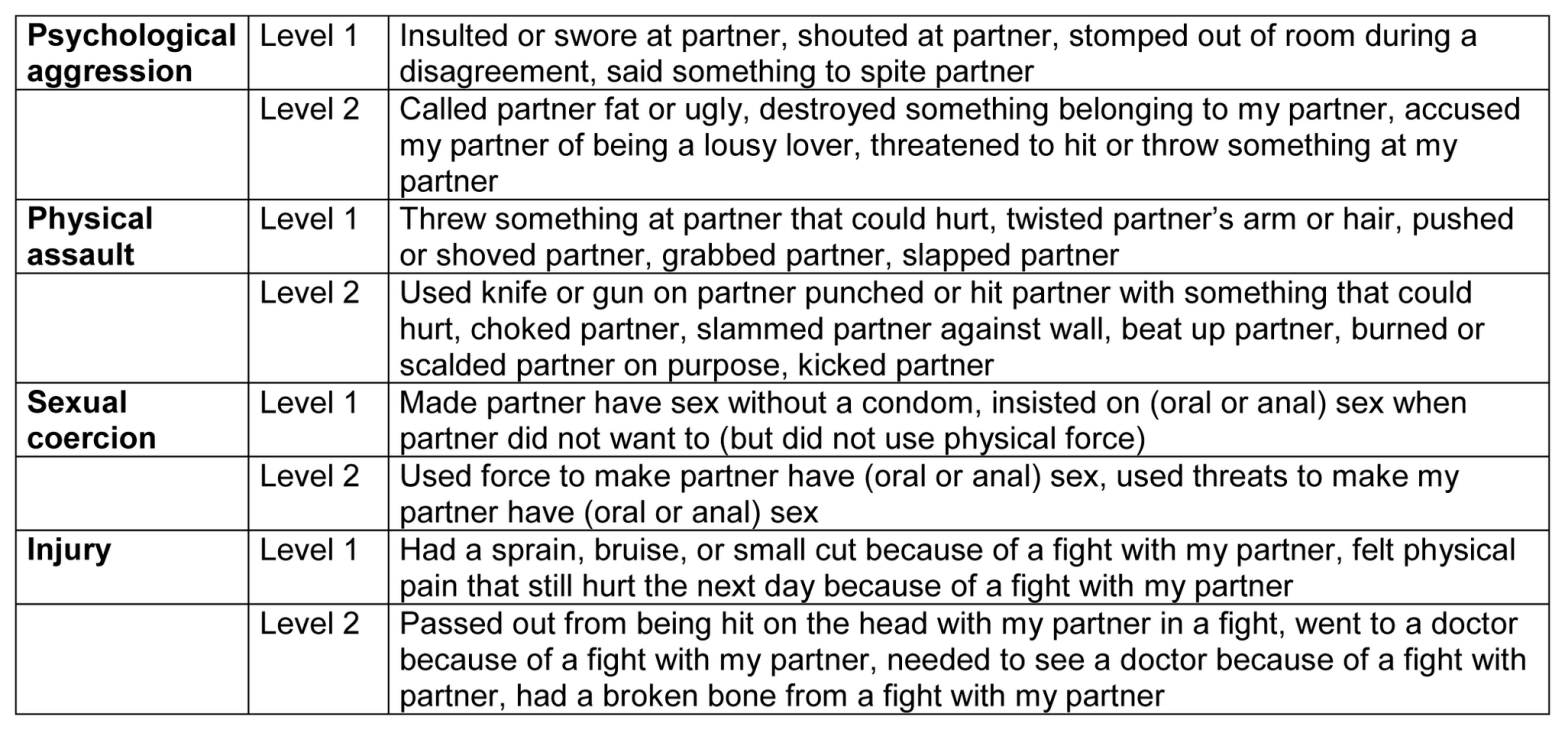
Items and subscales of the Revised Conflict Tactics Scale.

Interviewers collected demographic information including age, ethnicity and education level at 16 to 28 weeks of pregnancy (baseline)[22]. At this baseline, the interviewers used the Abuse Assessment Screen to measure physical and sexual violence in the past[29]. At 32 weeks of pregnancy and 24 months after birth, the revised Conflict Tactics Scale (CTS2) was used to measure prevalence of IPV victimization and perpetration[28]. The CTS2 questionnaire includes four scales: physical assault, psychological aggression, injury and sexual coercion. The CTS2 also takes into account the severity of violence (level 1 and level 2) as shown in Figure **1**. Annual prevalence was measured by indicating whether one or more of the acts in each scale were present in the past year. The variable “Combination of IPV forms” indicates whether more than one form of violence (psychological, physical, sexual violence and injury) was present [28,30]. Interviewers did not administer the CTS2 at baseline because the CTS2 measures IPV during a current or most recent relationship rather than relationships in the past. 

### Power Calculation

The sample size calculation was based on finding an effect in smoking reduction or cessation at the time of birth, at 12 months and at 24 months postpartum and was based on findings from the effects of the NFP study [15]. To detect an average improvement or a decrease in smoking by four cigarettes a day with a standard deviation of eight cigarettes, a power of 80% and an alpha of 5% were used. This resulted in a sample size of 57. Given that 25% of all women smoke at the start of pregnancy in the Netherlands, 228 participants in the control group and 228 participants in the intervention group were needed to detect a statistically significant effect.

### Statistical analyses

Data were analyzed with the statistical package SPSS 15.0 for Windows. Multivariable logistic regression analyses were performed to compare differences in dichotomous outcomes between the control and intervention groups. Numbers needed to treat (NNT) and odds-ratios and their corresponding 95% confidence intervals were calculated. NNT is defined as the estimated number of participants who need to be treated with VoorZorg rather than the usual care for one additional participant to benefit over a time period of two years[31]. Multivariable Linear regression analyses were used to compare continuous outcomes. 

We applied additional imputation techniques only for data from 24 months after birth because a completers/non-completers analysis was performed and indicated that we had enough data at 32 weeks for our analyses. In the control group, 110 CTS2 questionnaires were completed at T=32 weeks and 74 at T=24 months. Of these, 36 observations were carried forward, which is 33%. In the intervention group, 156 CTS2 questionnaires were completed at T=32 weeks and 110 at T=24 months. Of these, 29 observations were carried forward, which is 30%. First, we analyzed the data from 24 months after birth without imputation techniques. Then, last observation carried forward analyses and multiple imputation (MI) analyses were applied to impute missing values with Stata 12 (Stata Statistical Software: Release 12.0. College Station, Tex: Stata Corp;2001). Only results from the MI analyses were reported because this procedure results in more power, generates valid missing value imputations under a variety of missing data scenarios and is currently the most recommended missing data method. 

Multilevel Regression Analysis (MLwiN 2.24, Centre for Multilevel Modelling, Bristol, UK) was performed to measure the longitudinal relationship between the VoorZorg intervention and IPV victimization or perpetration. The dependency in the outcome variable being victim or perpetrator within the same person due to repeated measurements over time was accounted for by using multilevel models. In these models the time and intervention variable are included. Furthermore, the increase or decrease of the intervention effect over time was studied by introducing interaction terms between the intervention and time variable. Differences were considered significant if p-values were <0.05 (2-sided). All analyses were adjusted for possible confounders and effect modifiers.

 Attrition analysis was conducted to evaluate the differences on baseline characteristics and lifetime prevalence of IPV between participants who remained in the study versus those who did not.

## Results

### Baseline characteristics

**Figure 2:**
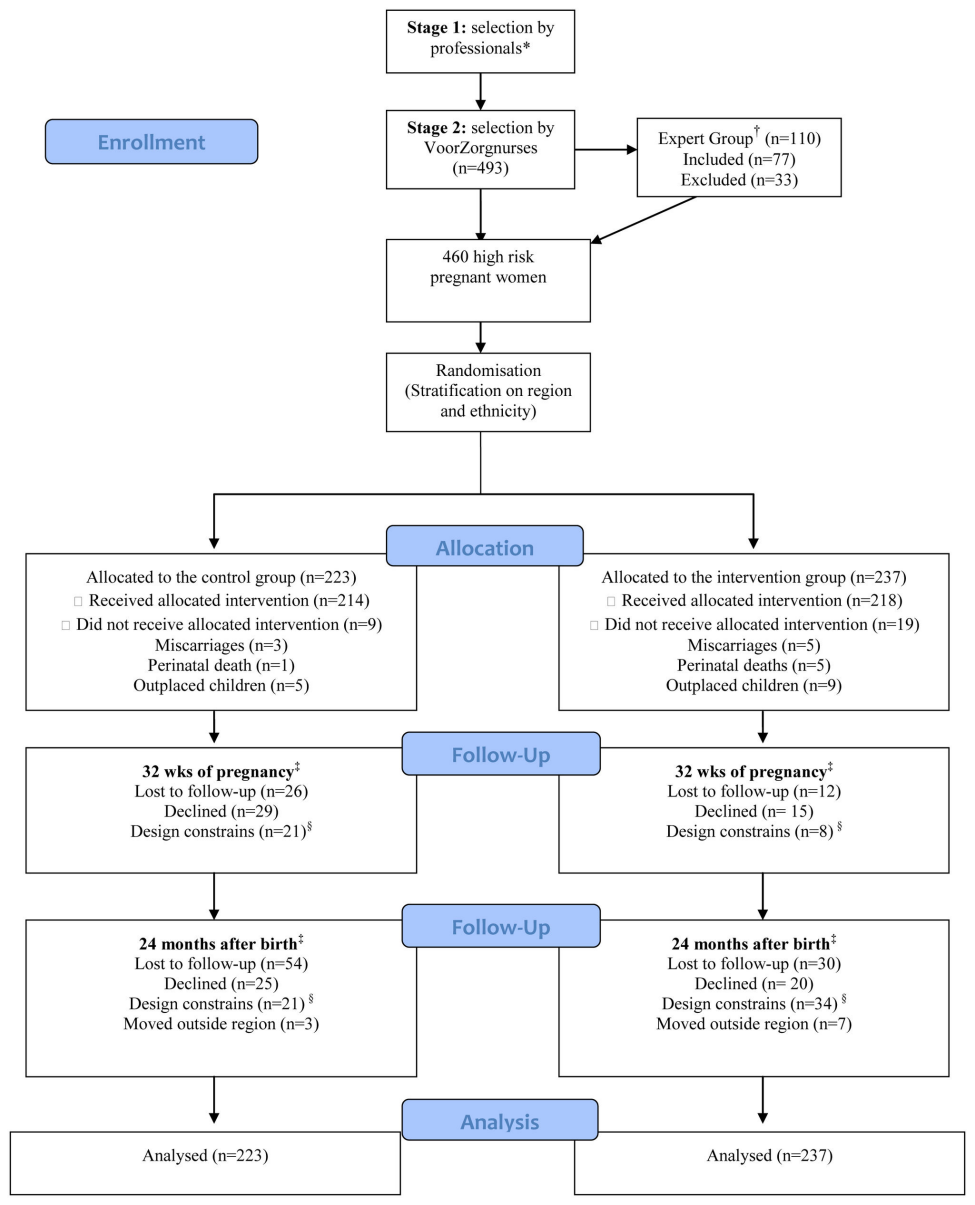
Flow of the participants through the study. *General practitioners, gynecologists, midwives, street corner workers (comparable to social workers) etc. The number of women for stage 1 is unknown; the pilot studies indicate that the VoorZorg nurses selected approximately 50% of them. ^†^ Only VoorZorg nurses could refer to the expert group, which settles arguments around inclusion . ^§^ No interviewer available, start-up problems RCT. **^‡^** Numbers were only used for the 24-month analyses with imputated values.

The flow of participants throughout the study is shown in Figure **2**. There were no significant differences in the reasons for loss to follow-up between the two groups. In the control group, 214 of the 223 participants received the allocated condition, 26 were lost to follow-up, 29 did not want to participate in the measurement of T=32 weeks, and 21 did not participate due to design constraints. Of the 138 measurements, there were 110 complete CTS2 questionnaires. In the intervention group, 218 of the 237 participants received the allocated condition; 12 were lost to follow-up, 15 did not want to participate in the measurement of T=32 weeks, and eight did not participate due to design constraints. Of the 185 measurements, there were 156 complete CTS2 questionnaires. Table **1** describes baseline characteristics. No significant differences in demographic characteristics or in the number of risk factors between the control and intervention groups were found at baseline. Of the two groups, 18% (n=40) of women in the control group and 19% (n=46) in the intervention group were physically abused during the past year, and 4% (n=9) in the control group and 5% (n=12) in the intervention group were sexually abused. Attrition analysis showed that participants who were lost to follow-up did not differ significantly from participants who remained in the study with regard to baseline characteristics displayed in table 1.

**Table 1:** Baseline characteristics of participants.

	**Control (n=223)**	**Intervention (n=237)**
**Mean age, years mean(sd)**	19.2 (2.6)	19.5 (2.8)
**Weeks of gestation mean(sd)**	19.6 (5.9)	20.1 (6.5)
**Region**		
**Urban**	147 (66)	158 (67)
**Rural**	76 (34)	79 (33)
**Ethnicity**		
**Dutch**	110 (49)	115 (49)
**Turkish/Moroccan**	13 (6)	13 (6)
**Surinamese/Antillean**	58 (26)	64 (27)
**Other**	42 (19)	45 (19)
**Education level**		
**Primary school**	7 (5)	11 (6)
**Pre-vocational Secondary education**	150 (96)	179 (94)
**Married/living together**	36 (16)	46 (19)
**Having a boyfriend**	49 (22)	70 (30)
**Living with boyfriend**	40 (18)	58 (24)
**Lifetime prevalence of IPV**	74 (33)	84 (35)
**Victim of physical abuse during past year**	40(18)	46(19)
**Victim of sexual abuse during past year**	9(4)	12(5)

Note. The information in Table 1 describes only those participants for whom data were available. Numbers are n (%) unless noted otherwise.

### Intervention delivery

The intervention is a structured program, in which the frequency of home visits is greater in the beginning during pregnancy and the first months after delivery. Women are included in the program at 20 ± 6 (mean ± SD) weeks of pregnancy. The number of home visits during pregnancy was 9 ± 4 (mean ± SD). Only two women were included late and gave birth early, in which case they received less home visits during pregnancy. There were no women included after 28 weeks of pregnancy. The majority of participants received between six and 13 home visits during pregnancy. 

## IPV

### Participant is victim

At 32 weeks of pregnancy, all participants reported experiencing level 1 psychological aggression, as shown in table **2**. Reports of level 2 psychological aggression were significantly lower in the intervention group than in the control group (OR 0.55; 95% CI 0.32 to 0.94). Significantly fewer women in the intervention group experienced level 1 physical assault (OR 0.38; 95% CI 0.22 to 0.66) and level 2 assault (OR 0.57; 95% CI 0.32 to 0.99). Experiences of level 1 sexual coercion were significantly lower in the intervention group (OR 0.47; 95% CI 0.19 to 0.90). The prevalence of level 2 sexual coercion (OR 1.09; 95% CI 0.41 to 2.92) and the prevalence of injuries experienced after a fight (OR 1.13; 95% CI 0.36 to 3.56) did not differ significantly in both groups. Significantly fewer participants in the intervention group were victims of more than two forms of violence compared with participants in the control group (OR 0.49; 95% CI 0.28 to 0.86). 

**Table 2:** Prevalence of IPV by treatment condition at 32 weeks of pregnancy and 24 months after birth.

**Participant is victim**	**Control %(n**)** (*n=110***)	**Intervention %(n) (*n=156*)**	**NNT**	**Odds ratio (95% CI)**
***32weeksofpregnancy***				
**Psychological Aggression^#^**				
**Level 1**	100% (110)	100% (156)	-	-
**Level 2**	56% (61)	39% (61)	6	0.55 (0.32 to 0.94)**^1^
**Physical assault**				
**Level 1**	58% (64)	40% (62)	6	0.38 (0.22 to 0.66)****^2^
**Level 2**	31% (34)	20% (31)	9	0.57 (0.32 to 0.99)**
**Sexual coercion**				
**Level 1**	16% (18)	8% (12)	13	0.47 (0.19 to 0.90)**
**Level 2**	6% (7)	7% (11)	-	1.09 (0.41 to 2.92)
**Injury**				
**Level 1**	26% (28)	16% (25)	10	0.57(0.31 to 1.05)
**Level 2**	5% (5)	5% (8)	-	1.13 (0.36 to 3.56)
**Combination of IPV forms**				
**mean** ^†^	1.9; 1.06	1.7; 0.96		-0.07 to 0.42
**>2 forms**	31% (35)	19% (29)	8	0.49 (0.28 to 0.86)**
***24 months after birth***	(***n=223***)	(***n=237***)		
**Psychological aggression**				
**Level 1**	73% (162)	74% (175)	-	0.99 (0.50 to 1.95)
**Level 2**	47% (105)	35% (83)	8	0.63 (0.34 to 1.14)
**Physical assault**				
**Level 1**	44% (98)	26% (62)	6	0.46 (0.24 to 0.89)**
**Level 2**	25% (56)	17% (40)	12.5	0.63 (0.29 to 1.39)
**Sexual coercion**				
**Level 1**	15% (33)	8% (19)	14	0.49 (0.19 to 1.27)
**Level 2**	5% (11)	8% (19)	-	1.61 (0.38 to 6.68)
**Injury**				
**Level 1**	23% (51)	16% (38)	14	0.63 (0.25 to 1.56)
**Level 2**	9% (20)	2% (8)	14	0.22 (0.03 to 1.57)
**Combination of IPV forms**				
**mean** ^†^	1.6;0.19	1.3;0.12		0.32 (-0.70 to 0.06)
**>2 forms**	36% (80)	23% (55)	8	0.51 (0.21 to 1.25)

Note. Multiple imputation analysis was conducted at 24 months after birth

NNT = numbers needed to treat over a 2-year time period

^†^ Numbers are presented as the mean; Standard deviation

** p<0.05; *** p<0.005; **** p<0.001

^#^ For explanation of levels 1 and 2: see Measurements in the Methods section

^1^ Adjusted for age and number of Sexually transmitted disease (STD) treatments

^2^ Adjusted for number of risk factors at baseline and number of STD treatments.

At 24 months after birth, multiple imputation analysis revealed that the prevalence of level 2 physical assault was significantly lower among women in the intervention group (OR 0.46; 95% CI 0.24 to 0.89). Other forms of violence were not significantly different at 24 months. 

Multilevel logistic regression analyses revealed that reports of level 2 psychological aggression and level 1 physical assault among women in the intervention group reduced significantly more over the course of the intervention than reports among women in the control group. 

### Participant is perpetrator

The percentage of participants who reported abusing their partner is illustrated in table **3**. At 32 weeks of pregnancy, participants in the intervention group reported using significantly less level 2 psychological aggression (OR 0.57; 95% CI 0.34 to 0.95) and level 1 physical assault (OR 0.57; 95% CI 0.34 to 0.95), and inflicted significantly less level 1 injuries to their partners (OR 0.53; 95% CI 0.29 to 0.96) than participants in the control group. Sexual coercion was the least common form of violence used by participants in both groups. Significantly fewer participants in the intervention group used more than two forms of violence towards their partner compared with participants in the control group (OR 0.53; 95% CI 0.30 to 0.94).

**Table 3:** Prevalence of IPV by treatment condition at 32 weeks of pregnancy and 24 months after birth when participant is perpetrator.

**Participant is perpetrator**	**Control %(n) (*n=110*)**	**Intervention %(n) (n=156)**	**NNT**	**Odds ratio (95% CI)**
***32wksofpregnancy***				
**Psychological aggression^#^**				
**Level 1**	87% (95)	89% (139)	-	1.59 (0.69 to 3.62)
**Level 2**	60% (66)	46% (72)	7	0.57 (0.35 to 0.94)**
**Physical assault**				
**Level 1**	65% (71)	52% (81)	8	0.57 (0.34 to 0.95)**
**Level 2**	33% (36)	28% (43)	20	0.78 (0.46 to 1.33)
**Sexual coercion**				
**Level 1**	6% (7)	7% (11)	-	1.10 (0.42 to 2.95)
**Level 2**	3% (3)	1% (2)	50	0.47 (0.08 to 2.84)
**Injury**				
**Level 1**	27% (30)	17% (26)	10	0.53 (0.29 to 0.96)**
**Level 2**	8% (9)	10% (15)	-	1.19 (0.51 to 2.85)
**Combination of IPV forms**				
**mean** ^†^	2.0; 0.97	1.7; 0.90		0.06 to 0.52**
**>2 forms**	31% (34)	19% (30)	8	0.53 (0.30 to 0.94)**
***24monthsafterbirth***	(***n=223***)	(***n=237***)		
**Psychological aggression**				
**Level 1**	80% (178)	76% (180)	25	0.89 (0.38 to 2.09)
**Level 2**	39% (87)	38% (90)	100	0.97 (0.50 to 1.89)
**Physical assault**				
**Level 1**	48% (107)	33% (78)	7	0.54 (0.28 to 1.03)
**Level 2**	25% (56)	14% (33)	9	0.48 (0.22 to 1.05)
**Sexual coercion**				
**Level 1**	18% (40)	3% (7)	7	0.10 (0.02 to 0.56)***
**Level 2**	5% (11)	3% (7)	50	0.60 (0.06 to 6.16)
**Injury**				
**Level 1**	24% (54)	17% (40)	14	0.63 (0.28 to 1.43)
**Level 2**	9% (20)	8% (19)	100	0.81 (0.29 to 2.31)
**Combination of IPV forms**				
**mean** ^†^	1.7;0.16	1.3;0.1	-	0.40 (-0.07 to -0.03)**
**>2 forms**	33% (74)	21% (50)	8	0.56 (0.25 to 1.28)

Note. Multiple imputation analysis was conducted at 24 months after birth

NNT = numbers needed to treat over a 2-year time period

† Numbers are presented as the mean; Standard deviation

** p<0.05; *** p<0.005

# For explanation of levels 1 and 2: see Measurements in the Methods section.

 Multiple imputation analyses revealed that at 24 months after birth, the prevalence of level 1 sexual coercion was significantly lower in the intervention group than in the control group (OR 0.10; 95% CI 0.02 to 0.56). The prevalence of psychological aggression, physical assault, level 2 sexual coercion, and injuries inflicted on partner were similar in both groups.

Multilevel logistic regression analyses showed that level 1 physical assault decreased significantly over time among participants in the intervention group and was significantly lower than in the control group. 

### Participant is both victim and perpetrator

The majority of the victims of psychological aggression or physical assault reported perpetrating abuse as well (approximately 85%). At 32 weeks of pregnancy, the prevalence of women being both victim and perpetrator was significantly lower in the intervention group for level 2 psychological aggression (OR 0.48; 95% CI 0.29 to 0.80) and level 1 physical assault (OR 0.44; 95% CI 0.27 to 0.72), as compared with the prevalence within the control group. At 24 months after birth, women in the intervention group had a statistically significant lower odds of being both victim and perpetrator of level 1 physical assault compared with women in the control group (OR 0.48; 95% CI 0.25 to 0. 93).

## Discussion

The current study shows that the VoorZorg program is effective in reducing victimization and perpetration of self-reported IPV during pregnancy and two years after birth among low-educated pregnant young women. Through nurse home visits, a trusting relationship is established between patient and nurse. By addressing factors that may increase the risk for IPV in general (e.g., a reduction in stress), as well as factors that may increase the risk for IPV in relation to a specific person (e.g., identifying an abusive relationship), victimization and perpetration due to IPV was significantly lower during pregnancy and two years after birth in a sample of low-educated pregnant women compared with women in a control group. The reduction in IPV means that an important risk factor for compromised fetal development is mitigated through these nurse home visits. Furthermore, because the program works to proactively prevent IPV, it may have longer-term positive health effects on parents and their children[32]. VoorZorg is the Dutch equivalent of the NFP, which is widely recognized as an evidence-based preventive intervention that targets child abuse and neglect in the United States. We are the first researchers outside of the US to assess the effect of the NFP and to report the results of an RCT studying the effectiveness of VoorZorg on children’s health and development.

Olds and other researchers have found that the NFP is associated with many positive and long-lasting effects of the NFP on mother and child development. Our findings regarding the positive effects that the program has on IPV victimization and perpetration among young pregnant mothers adds to this evidence. However, Olds et al. did not find that the NFP has an effect on IPV during pregnancy and after birth[18]. Some researchers have argued that participants are reluctant to report violence in families with young children because nurses in the United States are obliged mandated to report child abuse and participants might risk to lose losing their child to Child Protection Services[33]. In the Netherlands, nurses are not required to report child abuse, which may explain the differences between our findings and previous studies. Another explanation might be that, in the Netherlands, it is more socially accepted to speak about non-marital sex and IPV than in the US[34]. Therefore, VoorZorg nurses can address IPV more effectively. Eckenrode et al. showed that the presence of IPV moderated the impact of the program on the prevention of child abuse and neglect[20] with smaller effects on abuse and neglect at higher levels of IPV. The current study reveals that home visits reduce incidents of intimate partner violence in a sample of high-risk young pregnant women; future analyses will reveal whether an additional impact on child abuse and neglect will be found which should be in line with the findings of Eckenrode et al.

In this high-risk sample of low-educated young pregnant women, 100% reported experiencing psychological violence, 58% reported experiencing physical violence, 26% reported experiencing injuries after a fight and 16% experienced sexual violence during pregnancy. All women in this sample reported experiencing psychological violence, which indicates that it may well be a situational couple violence which may be present in many couples relationships[35]. Similarly, our study found that women reported high levels of psychological aggression at 24 months. A potential explanation for this finding is that the mothers experienced a high level of stress because the baby developed into a toddler and needs much more attention. Toddlers want to explore their limits through new experiences and are therefore more prone to encountering hazards, such as falling on the stairs. For the high-risk young mothers in this study, this stage in their child’s development is a very stressful period. This is exacerbated by the fact that their children often exhibit more externalizing behavior and listen less carefully compared with children in families where there is more structure and calm. Mc Farlane et al. reported that 17% of low income, pregnant women experienced physical or sexual violence [29]. The prevalence of these forms of abuse reported in the current study are also high, even when compared with pregnant adolescents living below the poverty level, who already are at an elevated risk of experiencing violence (21%)[36]. Moreover, a high percentage of women (69%) in the VoorZorg study revealed a history of violence earlier in life (unpublished data). These findings emphasize the importance for health care workers to focus on this vulnerable group. In addition to the effect that violence has on a mother, a child that grows up in a violent environment is more likely become involved in a violent relationship later in life[37]. It is important to break this cycle of violence.

 A major benefit of home-visiting interventions is that they succeed in reaching high-risk young pregnant women, who are notoriously hard to reach for regular services, during a vulnerable stage in life over a prolonged period of time. In our study, female participants received between six and 13 home visits during pregnancy. These home visits were standardized and the intervention delivery was comparable with the work of Olds et al. in terms of timing and intensity of the home visits. The intervention successfully addresses multiple risk factors that can compromise the development of a young mother and child. As a result, participants receive a myriad of benefits; mothers become better able to raise their child, and children become less exposed to stress[38]. By reducing a child’s exposure to violence and thereby reducing exposure to stress, the child’s brain development can improve. An elevated cortisol level, a hormone released during stress, can affect brain development and can lead to conduct disorders, increasing the risk of having stress-related psychiatric disorders in later life[39]. Women receiving the VoorZorg program reported using less physical violence towards their partner than women receiving the usual care. Generally, less attention is paid to female perpetrators and almost no evidence exists about effective strategies to reduce female perpetration of physical violence[40]. In the current study, we did not measure the reasons that women use violence, but previous studies have suggested that women use physical force because they feel emotionally hurt and want to express their feelings[40]. This suggests that women receiving the VoorZorg program have developed a different strategy for expressing themselves rather than using violence. Women also use violence to defend themselves, which may imply that women receiving the VoorZorg program feel less threatened by their partners[41]. According to Johnson’s typologies IPV, our study population could be categorized as “violent resistant”, which means that the woman is violent but her partner is both violent and controlling. This form of violence is almost exclusively common among women[35,42]. Future research should determine what causes women to use physical force and the mechanisms by which VoorZorg reduces their use of force, taking in account that many women are “violent resistant”. 

This study also reveals some key factors that intervention programs should integrate to reduce IPV and its impact on health. Firstly, it is important to screen at-risk groups and address IPV risk factors, such as alcohol consumption and financial dependency. Home visiting programs seem to be promising in addressing these risk factors. Nurses see the home environment and can detect risk factors for IPV and observe whether IPV is present[43]. Secondly, an important factor is creating an open and nonjudgmental dialogue between nurse and patient to make it easier for women to admit that IPV is present[44]. Nicolaidis et al. showed that victims of abuse find it very important that their relationship with health care professionals is based on trust and respect[45]. When patients have a trusting and respectful relationship with their health care provider, a safe space is created in which women can speak about their experiences with violence. Thirdly, when IPV is present in a family it’s important to reduce its impact. Social support and an increase in one’s self-efficacy appear to have a substantial effect on reducing the impact of IPV[46]. Lastly, it is important to address violent and controlling behavior among perpetrators, when perpetrators use controlling behavior, their partners may feel forced to use violence. 

A strength of this study is that VoorZorg is designed for low-educated, young pregnant women. This young population requires special attention because they have multiple risk factors associated with IPV and many of them do not see violence as a reason to end their relationship[47]. IPV could therefore have a greater impact on their life compared with adult women. Given our results, we expect that many women in our study population have post-traumatic stress disorder (PTSD) because of current and past IPV. VoorZorg does not, however, focus on treating PTSD. Women with PTSD complaints are referred to mental health services when possible. Another limitation of this study was the high loss to follow-up at the 24 month measurement as this could limit the generalizability and the integrity of our results. Two main reasons for loss to follow-up are: 1) women, especially in the control group, were not traceable and 2) participants declined to participate in the interviews despite informed consent. To diminish these problems, the researchers instructed the interviewers on how to address untraceable women. Methods included using social media and youth health care organizations or General Practitioners to restore the contact and obtain the most recent contact information. Child Health Care professionals and GP’s often could not trace women in the control group, which underscores their inaccessibility. Because the VoorZorg nurse regularly visited women in the intervention group, they were easier to contact throughout the study. Another limitation was the use of self-report questionnaires to measure IPV. Self-reports were not confirmed by other informants. The CTS2 is criticized because of methodological shortcomings, such as not addressing coercion, control or motives, and no measurement of the different types of partner violence[30,48]. However, we used the CTS2 because we wanted to measure the frequency and nature of the different types of IPV. We also use the CTS2 because it is the most widely used instrument and has been successfully used to identify intervention effects in other studies about IPV [21,49]. We measured the context and consequences of IPV with other variables . 

We recommend that further research examine how to decrease loss to follow-up among low-income pregnant young women. We also recommend that future interventions should address IPV perpetration by women. Care providers should be aware that perpetration is prevalent among high-risk women and should address the reasons that women use violence. For violence, even if it is for self-defense, has many health consequences for the parents and their children. 

## Conclusion

Overall, both the control and intervention group reported a high prevalence violence during pregnancy. At 32 weeks of pregnancy, significantly fewer women in the intervention group were violated by their partner and used significantly less violence toward their partner. At 24 months after birth, women receiving home visits experienced less physical violence. They also used less sexual violence towards their partner. In conclusion, the VoorZorg intervention is effective in reducing IPV during pregnancy and up to two years after birth. Further research is needed to investigate the long-term effects of VoorZorg on IPV and children’s development. 

## Supporting Information

Adjustments in the Dutch Version of Infancy Guidelines S1 (DOC)

Adjustments in the Dutch Version of Pregnancy Guidelines S1 (DOC)

Checklist S1 CONSORT Checklist.
(DOC)

Protocol S1 Trial Protocol.
(PDF)
