# Supplementary material for: Effect of Nurse Home Visits vs. Usual Care on Reducing Intimate Partner Violence in Young High-Risk Pregnant Women: A Randomized Controlled Trial
Source: PLoS One. 2013 Oct 21;8(10):e78185. doi: 10.1371/journal.pone.0078185 (PMC3804627; doi:10.1371/journal.pone.0078185)
Supplement: Adjustments in the Dutch Version of Infancy Guidelines S1 — (DOC) [file pone.0078185.s001.doc]

**Adjustments in the Dutch version of Infancy Guidelines as compared to the original NFP version S1**

*The translation of the NFP Infancy Guidelines to the Dutch VoorZorg Baby Handleiding is based on the Infancy guidelines, Fourth Printing, 2000, rev. 04/2001 & 10/2002.*

General information: we had a group of commentators who read the first (literal) translation of the Guidelines, consisting of 4 very experienced nurses, working in different organizations for public child health care. In this group were also two developmental psychologists with experience in working with baby’s and/or young mothers, and a midwife with educational experience. Their comments lead us to make several adjustments, mentioned below, resulting in the version of the Baby Handleiding that thereupon was used by the VoorZorg nurses.

1. **Attuning to services of midwife and maternity care**

*Adjustment:* During the first visits we give attention to the role of the midwife and ‘Maternity Caretaker’ during the first week after delivery and how the nurse can tune in and complement her activities to the services of these professionals.

*Reason:* Besides the care of the midwife during the first period after the birth of the child, maternity care is an important specialized form of care in the Netherlands for mothers who recently gave birth. Maternity care is in principle given the first eight days after delivery of the baby; from 8 hours a day during the first days to 5 hours during the last few days. There are other variations possible. Besides, the maternity nurse can be present at childbirth and assist the midwife.

1. **Attuning to services of regular public child health care (consultation bureaus)**

*Adjustment:* We pay attention throughout the guidelines that the activities of the home visiting nurse has ‘added value’ to other regular youth health care.

*Reason:* Avoid overlap and take care that the program complements regular care.

E.g. we adjusted some of the moments the ‘Bright Futures’ facilitators are discussed and adapted some of its content to the services of the consultation bureaus.

*Reason:* The so-called ‘contact moments’ the mother and child have with the public child health care system are sometimes different from those in the Infancy Guidelines. We followed the official standards for contact moments in Dutch Public Child Health Care.

1. **NCAST and PIPE**

*Adjustment:* The materials of NCAST and PIPE have not been included.

*Reason:* We do not have the licenses to use NCAST and PIPE and/or their original materials.

We have worked at replacing these materials as follows:

- The NCAST leaflets will be replaced by other materials and methods that will cover the topics in question. As far as educational materials are concerned, there are several good materials (written, and audio-visual) that consultation bureaus for public child health care in the Netherlands use concerning (breast)feeding, infant crying, sleeping patterns, responsive mother-child interaction, fostering of social-emotional and cognitive growth etc.

We have given the nurses a list of these materials, and refer to them in the guidelines. One of these materials is the so-called ‘Groeiboek’ (*Growth book*). This booklet is given to every parent that visits the consultation bureau for youth health care. It can be offered in several different languages. The book contains concise information on many topics concerning the physical and psycho-social development of children during the first 4 years of life, and the role of parents.

- As far as PIPE topics are concerned, we have followed two ways:

First, we kept the topics as such mentioned in the Babyhandleiding, although we do not have the original manuals and materials. Still, we think the nurse can give attention to the mentioned topics, and where possible we refer to relevant educational materials for the parent (e.g. the Groeiboek). To give just one example: in I-8 under Maternal Role, PIPE-topic ‘Baby Cues’ is mentioned, to stimulate skills to read and respond to baby’s emotional signals. In our translation the nurse is asked to ask the mother if and how she can notice what goes on inside her baby, and to give the mother information how she can read and respond to her baby’s cues. Than we refer to a certain page in the Groeiboek, which is about ‘What does your baby want?’. The nurse is than to discuss this topic with the mother. We realize this is not a complete and full replacement of the PIPE module, but we feel that this is better than leaving the PIPE topics completely out. Public Child Health Care nurses are experienced in supporting parents on these topics by their regular training and work.

A second way we have worked at an alternative for PIPE is (after analyzing several available programs in the Netherlands) by integrating Video Hometraining in the program. A scientific review of this intervention by Fukkink will appear in September 2006. VoorZorg-nurses are currently receiving an extensive training and supervision in this method. Information on this method is given to prof. Olds through articles and a demonstration of its use in VoorZorg is given during his visit to the Netherlands in June 2006.

VHT will be integrated into the program. Video takes of mother-child interactions will be made at the following moments:

- First year of life: Four video takes: first month, 8th month, 12th month
- Second year of life: Two video takes: 15^th^ month, 18^th^ month.

1. **Breastfeeding**

*Adjustment:* More information on breastfeeding en emphasis on the advantages of breastfeeding throughout the guidelines, e.g. by referring to specific educational material on this topic.

*Reason:* This is in line with the current professional views and working standards in the Netherlands.

1. **Safety**

*Adjustment:* We have given attention to safety-related topics that are specific for the Dutch context. E.g. and more attention for traveling by bicycle. Also adhering to European norms and laws concerning child-safety.

There are very good materials, the so-called ‘Veiligheidskaarten’ (*Safety Cards*), that are especially written for parents of children 0-6 months, 6-12 months, 1-2 years and 2-4 years of age. These cards have proven to be effective (research by EUR Rotterdam). Besides, much information can be found of a specialized website [www.veiligheid.nl](http://www.veiligheid.nl/), to which we refer throughout the guidelines.

1. **Growth and development: Ages and Stages Questionnaires**

Adjustment: We used translations in Dutch of the ASQ at 6 and 12 months, in stead of 4, 6 and 8 months.

Reason: this questionnaire is not used and practically unknown in the Netherlands. We found a researcher at the University of Amsterdam who has translated and used on an experimental basis the versions for 6, 12, 18 and 24 months. We will monitor how these questionnaires can be used in the context of VoorZorg. As alternatives for the ASQ at 4 and 8 months, we refer to short checklists in the ‘Groeiboek’ by which parents can give information on the development of their child.

Also, in the regular Youth Health Care for 0-4 yr olds, a observational monitoring system for psycho-motor development is used throughout the country. This is called the ‘Van Wiechen schema’. This is a well developed and validated instrument/method by which growth and development of the child is monitored and abnormalities can be detected in an early phase.

1. **Smart Choices = 'Slim aanpakken'**

*Adjustment:* We have translated only one of the two versions (version one). Names of persons have been changed to common Dutch names.

*Reason:* At this experimental stage we first want to find out whether this module will make sense in working with clients in the Dutch context. The version with the most simple use of language probably will serve this purpose best. Besides, we find the differences between the two versions to be very small.

We have reviewed other possible modules that have the same purposes and qualities as Smart Choices, but have already been tested in the Netherlands. However, so far we have not found a good alternative.

1. **Design and illustrations**

*Adjustment:* Adaptation of the design, and especially of the educational materials and facilitators.

*Reason:* As we started with the translation of the Pregnancy guidelines, we did not have the original design in Quark. Therefore we decided to make a new lay out which had mostly consequences for the illustrated materials (educational and facilitators). For reasons of uniformity we have used the same lay out for the Baby handleiding. We hope to make a nicer illustrated version in the future.

**Specific adjustments**

- We left out the instructions and material concerning lead poisoning. Probably due to the use of other materials for water supplies, this is not considered a health threat in the Netherlands.
- Attention is given that mothers know about, and if possible follow, regular courses that are being offered, e.g. for baby massage, doing exercises after pregnancy, meetings for young mothers and fathers, etc.
- Where necessary we adapted materials on day care to how these services are arranged in the Netherlands (e.g. concerning norms for safety and quality).
- Where appropriate, we adapted teachings and materials on food and feeding to generally accepted and ‘state of the art’ education and materials in use in the Netherlands (e.g. the dietary review).
- Concerning the subject of domestic violence, a reference is made to recent booklets, a.o. by the Ministry of Justice, that gives advice to women who are abused by their partners.
- Throughout the guidelines, wherever possible and appropriate, we have included references to informative websites, to complement educational materials.

In general, our group of commentators was very positive on the form and content of the program, as described in the Handleiding. At some points remarks were made about the fact that the educational materials require a lot of reading and may be boring in their lay out (sometimes a lot of reading and no illustrations). There also was doubt whether materials and facilitators would be ‘too much’. In the literal sense of the word, but also in the sense that mothers might not be motivated to work with all these materials (especially the Smart Choices). On the other hand, the completeness of the guidelines was praised.

Klaas Kooijman,

13-06-2006
