# Supplementary material for: Effect of Nurse Home Visits vs. Usual Care on Reducing Intimate Partner Violence in Young High-Risk Pregnant Women: A Randomized Controlled Trial
Source: PLoS One. 2013 Oct 21;8(10):e78185. doi: 10.1371/journal.pone.0078185 (PMC3804627; doi:10.1371/journal.pone.0078185)
Supplement: Adjustments in the Dutch Version of Pregnancy Guidelines S1 — (DOC) [file pone.0078185.s002.doc]

**Adjustments in the Dutch version of Pregnancy Guidelines as compared to the original NFP version S1**

*The translation of the NFP Pregnancy Guidelines to the Dutch VoorZorg Zwangerschapshandleiding is based on the Pregnancy guidelines, Fourth Pinting, 2000, rev. 04/2001 & 10/2002.*

1. **Giving birth at home**

*Adjustment:* More emphasis on giving birth at home. Examples:

- An extra information sheet that discusses the choice between giving birth at home or at the hospital (P-6).
- An extra question has been added in facilitator ‘My baby and me’ as to whether the client has already made a decision on wanting to give birth at home or in the hospital.
- Information on procedures after delivery in P-10 has been adjusted to Dutch obstetric procedures
- Information on purchasing a so-called 'kraampakket (= 'maternity package'), the costs of which are often covered by health insurance, is included in P-10 and P11.
- Information about packing for the hospital (P-11) has been supplemented by an Information sheet ‘What do you need for giving birth to your baby at home?’(P-11), which among other things discusses topics like: ‘How can you get in touch with your midwife quickly, if needed?’, and ‘What materials do you need to have ready in your home?’
- Phrases like ‘After the mother returns from the hospital…’ are replaced by ‘After the delivery of the baby’…
- In P-13 once more the mother is reminded to call the midwife immediately as soon as true labor starts.

*Reason:* 85% of all pregnant women are being medically attended to by a midwife from the start. In total 50% of all deliveries are being attended to by a midwife (30% are done at home and 20% are done in the hospital under the guidance of a midwife). The other 50% of all deliveries take place in the hospital under the guidance of an obstetrician.

1. **Pregnancy classes**

*Adjustment:* The suggested client activity ‘Investigate prenatal classes’ is transferred from P-8 (starting from week 27-28) to P-3 and P-4 (starting from week 16). This subject is brought up again later (notably at P-6) to check if the client has indeed taken action to inscribe and enroll in a pregnancy class.

The instructions in P-6, under Nutrition and Exercise: ‘Explain …..’ and ‘Demonstrate…’ can be skipped if the client already follows pregnancy classes.

*Reason:* Pregnancy classes in the Netherlands start as a rule in the 5th or 6th month of pregnancy (from week 22), or even sooner. Registration has preferably to take place in the 4th month (between weeks 16 and 20).

1. **Maternity care**

*Adjustment:* Already at the first visit (P-1) the home visitor checks if the client has taken steps to arrange for maternity care. If not, in subsequent visits this subject is brought up again. In P-13, the tasks of the maternity nurse are (again) explained and the temporary transfer of certain duties of the home visitor to the maternity nurse are discussed.

*Reason:* Maternity care is an important form of care in the Netherlands for mothers who recently gave birth. Maternity care is in principle given the first eight days after delivery of the baby; from 8 hours a day during the first days to 5 hours during the last few days. There are other variations possible. Besides, the maternity nurse can be present at childbirth and assist the midwife.

Registration for maternity care has preferably to take place before the 4th month of pregnancy, because there is a shortage of maternity care in some parts of the country.

1. **Performing medical actions / overlap with duties of the midwife or obstetrician**

*Adjustment:* Instructions for performing medical actions (e.g. checking for the growth of the fetus, assessing the client breast, blood pressure, reflexes, weight and edema) are replaced by instructions to ask if the regular health care provider (midwife or obstetrician) has performed these actions, what the results were, whether the information given was clear to the client, discuss which consequences this may have for the health behavior of the client and how the visitor can be of help with attaining specific health goals.

*Reason:* Medical actions in the personal health domain are already routinely performed by the midwife or obstetrician, and are their exclusive professional responsibility. See e.g. P-1 and P-8.

1. **Overlap with duties of maternity nurse and youth health care**

*Adjustment:* Risk of ‘overlap’ will probably be also present in the ‘Infancy Guidelines’ as concerned the regular activities of the maternity nurse and the youth health care (doctor and nurse). We will therefore constantly be concerned that the activities of the home visiting nurse has ‘added value’ to other regular (health) care.

*Reason:* Avoid overlap and take care that the program complements regular care.

1. **Education about youth health care**

*Adjustment:* Information for the client is added about the content of youth health care, how to register, PKU-screening after the baby is born, first appointment at the well-baby clinic etc. (P-10).

*Reason:* This information is very important within the Dutch system of health care for baby and mother.

1. **Smoking during pregnancy**

*Adjustment:* In the instructions and training of the home visiting nurse a connection has been made with the activities of STIVORO / Stichting Volksgezondheid en Roken (Foundation Public Health and Smoking), a strong current government initiative to prevent and reduce the incidence of smoking. STIVORO has well-developed educational materials for pregnant women, midwifes, and parents with young children. These are incorporated in the guidelines (e.g. in P-3) as well as in the nurse-training.

*Reason:* The STIVORO materials and philosophy fit in very well with the program. The materials for mothers and professionals are well developed and already widely known.

1. **Ultrasound and prenatal screening**

*Adjustment:* The information about ultrasound and chromosome-screening is adjusted to current health care practices in the Netherlands. New educational materials have been made; see P-2.

*Reason:* All pregnant women in the Netherlands will have an ultrasound during the first three months of their pregnancy. Thus the duration of the pregnancy can be reliably measured, which is why this ultrasound is called a ‘term-echo’ in the Netherlands. A second ultrasound will be standard procedure at the 20th week of the pregnancy.

It is important that the home visitor has the necessary information available about the standard procedure of blood-screening by the midwife or obstetrician and about the different forms of prenatal screening for possible chromosome-deviations. Information on this subject can be found in the leaflet ‘Zwanger’ (= ‘Pregnant’), published by the Dutch Association for Obstetrics and Gynecology et al (2004).

1. **Access to files and exchange of information with other care providers**

*Adjustment:* Instructions for the home visitor to check the file of the regular care provider have been complemented by the phrase: ‘under the condition that the client has given permission to do so’ (e.g. in P-2). In P-1, the client is explicitly asked to sign a consent-form for the exchange of information with other care and service providers.

*Reason:* Under Dutch law it is not permitted, unless with expressed consent of the client, to exchange information about the client with others, look into files of other caregivers, or give others access to one’s own file about the client.

1. **Eating and drinking**

*Adjustment:* The food guide pyramid and the Nutritional guidelines (P-2) have been combined into one educational sheet ‘Eating and drinking during pregnancy’.

*Reason:* Making information more transparent and simple.

*Adjustment:* Information is added in P-2 to avoid certain ‘raw’ types of cheese, and the importance of taking folic acid and vitamin D.

*Reason:* This is standard education for pregnant woman in the Netherlands (see leaflet ‘Zwanger’= ‘Pregnant’, mentioned under #8).

*Adjustment:* Certain foods and drinks have been replaced in the Key foods checklist.

*Reason:* Adjustment to feeding habits and use of certain types of food in the Netherlands.

1. **NCAST and PIPE**

*Adjustment:* The materials of NCAST and PIPE have not been included.

*Reason:* We do not have the licenses to use NCAST and PIPE and/or their original materials.

We have worked at replacing these materials as follows:

- In P-2, the use of the NCAST Network survey is replaced by certain questions to the client, that summarize the survey: ‘Discuss with the client and her partner how important persons in their network could be of help to them, and how simple or troublesome it is to ask those persons for help if necessary’.
- The NCAST leaflets 1 to 5 will be replaced by other materials and methods that will cover the topics in question. As far as educational materials are concerned, there are several good materials (written, video and dvd) in the Netherlands concerning (breast)feeding, infant crying, sleeping patterns etc. For methods to foster mother-child interaction and attachment we have looked at several available programs. At this moment we think the best option (from the viewpoint of quality of the intervention, available training facilities and possibilities for incorporation in the program) is video-hometraining.

1. **Breastfeeding**

*Adjustment:* More information on breastfeeding en emphasis on the advantages of breastfeeding, particularly in P-6 and P-9. In P-13 the leaflet *‘Borstvoeding, 7 stappen voor het 1^e^ jaar’* (= ‘Breastfeeding, 7 steps for the first year’) is being discussed with the mother.

*Reason:* This is in line with the current professional views and working standards in the Netherlands.

1. **Smart Choices = 'Slim aanpakken'**

*Adjustment:* We have translated only one of the two versions (version one). Names of persons have been changed to common Dutch names.

*Reason:* At this experimental stage we first want to find out whether this module will make sense in working with clients in the Dutch context. The version with the most simple use of language probably will serve this purpose best. Besides, we find the differences between the two versions to be very small.

We have reviewed other possible modules that have the same purposes and qualities as Smart Choices, but have already been tested in the Netherlands. However, so far we have not found a good alternative.

1. **Design and illustrations**

*Adjustment:* Adaptation of the design, and especially of the educational materials and facilitators.

*Reason:* We did not have the original design in Quark. This had mostly consequences for the illustrated materials (educational and facilitators). As it would be too costly and time-consuming to make new illustrations for this experimental phase, we decided to use for now a more simple design and some simple illustrations when necessary. The only original illustrations are those of the Smart Choices lesson in P-8 (facial expressions), and slightly adapted versions of the clocks in P-3 ('Mothers typical day') and P-6 ('Exercise for health and fun').

**Specific adjustments**

- In visit 1, under Personal Health, point A1a: the phrase *'Explain that all information is confidential except in situations in which mandated reporting applies'*, is replaced by *'If the nurse thinks that she has to discuss information with other professionals, she will first discuss this with the client'*. E.g in case of suspected child abuse (for which there is no mandatory reporting in the Netherlands) , this will be discussed with the client and subsequently contact can be made with an Advisory and Reporting Point Child Abuse.
- The information for the client in Visit 1, under Greeting, point G, on how to act when the client has concerns or complaints, is adapted to the 'protocol for handling complaints' the agency already has in operation.
- In Visit 2, under Goal setting and negotiation for the next visit, point C, the instruction *'Review prenatal clinical record….. Note any risk factors that…. etc.* is replaced by: *'If the client has given signed consent for this in Visit 1, discuss with her regular care provider (if possible with reviewing the client's record) if they have knowledge of client-factors that are important for complementary education or guidance'.*
- In Visit 2, under Personal network relationships, a Teaching point is added that asks the client if she has already some first ideas whether she would like to have the baby in official child-daycare. It is important to think about this topic rather early and to register soon for this facility, because there may be long waiting-lists.
- In Visit 3 (and 2) some suggestions have been added on educational materials about the growth and development of the fetus during pregnancy, from some popular magazines that are specifically concerned with pregnancy. These materials contain good information and nice illustrations, with which it becomes easier for the home visitor to discuss this subject, and foster knowledge and mother-baby attachment.
- In Visit 3, concerning the subject of domestic violence, a reference is made to materials in the Infant Guidelines (p.273-277), and also to a recent booklet of the Dutch Ministry of Justice, that gives advice to women who are abused by their partners.

This reference is made again in Visit 13, where domestic violence is brought up again under Personal network relationships

- In Visit 5, facilitator / questionnaire 'How is it going between us?' (= 'Hoe gaat het tussen ons?'), two extra questions have been added, that use a negative phrasing. Question 10: 'You do not listen well to me', and question 18: 'You tell me too much how to do things'. As all other questions are put in a positive wording (except #7), in this way the client may have more options to express negative feelings.
- In Visit 7, facilitator 'My sexuality' (= 'Mijn seksleven') the questions about using a diaphragm have been moved up in position, because this method is hardly used in the Netherlands.
- In Visit 7, facilitator 'Using birth control' (= Gebruik van voorbehoedsmiddelen'), question 6, the names of contraceptives have been narrowed down to three categories: the pill, IUD (='spiraaltje'), and condom and/or geld. These are the types most often used in the Netherlands.

At question 1, if answered 'No', there is an instruction to skip to question #7, instead of #8.

- In Visit 8, the educational material about 'Baby's Name' is adapted to Dutch law. Also a reference is made to other educational material, e.g. the judicial rules concerning legitimating a child by its father outside marriage etc.
- In Visit 10 the educational material about circumcision of boys is adapted to the Dutch context. This practice is much less common in the Netherlands (estimated 10%) than in the USA (estimated 62%). Mostly done for medical reasons, or for religious reasons (Jewish, Islam: some days or weeks after birth). If done is a hospital for medical reasons, always under full narcosis.
- In Visit 10 the information on the safety rules for cribs and beds is adapted to Dutch / European law.
- In Visit 13 an educational sheet is added about 'Recovery of the body after giving birth', so that the client can read this information and ask for more information by the home visitor when needed.
- Visit 13 has an extra instruction item for the home visitor, to talk with the mother about the importance of keeping herself from starting to smoke again (when she has stopped during pregnancy) after giving birth. The earlier mentioned materials by STIVORO can be well used for this purpose.

Klaas Kooijman,

22-12-2004
