# Supplementary material for: Effect of Nurse Home Visits vs. Usual Care on Reducing Intimate Partner Violence in Young High-Risk Pregnant Women: A Randomized Controlled Trial
Source: PLoS One. 2013 Oct 21;8(10):e78185. doi: 10.1371/journal.pone.0078185 (PMC3804627; doi:10.1371/journal.pone.0078185)
Supplement: Protocol S1 — Trial Protocol. (PDF) [file pone.0078185.s004.pdf]

Mejdoubi et al. BMC Public Health 2011, 11:823
http://www.biomedcentral.com/1471-2458/11/823

BMC Public Health

STUDY PROTOCOL Open Access

Addressing risk factors for child abuse among high risk pregnant women: design of a randomised controlled trial of the nurse family partnership in Dutch preventive health care

Jamila Mejdoubi[1], Silvia van den Heijkant[1], Elle Struijf[2], Frank van Leerdam[1], Remy HiraSing[1] and Alfons Crijnen[3*]

Abstract

Background: Low socio-economic status combined with other risk factors affects a person's physical and psychosocial health from childhood to adulthood. The societal impact of these problems is huge, and the consequences carry on into the next generation(s). Although several studies show these consequences, only a few actually intervene on these issues. In the United States, the Nurse Family Partnership focuses on high risk pregnant women and their children. The main goal of this program is primary prevention of child abuse. The Netherlands is the first country outside the United States allowed to translate and culturally adapt the Nurse Family Partnership into VoorZorg. The aim of the present study is to assess whether VoorZorg is as effective in the Netherland as in the United States.

Methods: The study consists of three partly overlapping phases. Phase 1 was the translation and cultural adaptation of Nurse Family Partnership and the design of a two-stage selection procedure. Phase 2 was a pilot study to examine the conditions for implementation. Phase 3 is the randomized controlled trial of VoorZorg compared to the care as usual. Primary outcome measures were smoking cessation during pregnancy and after birth, birth outcomes, child development, child abuse and domestic violence. The secondary outcome measure was the number of risk factors present.

Discussion: This study shows that the Nurse Family Partnership was successfully translated and culturally adapted into the Dutch health care system and that this program fulfills the needs of high-risk pregnant women. We hypothesize that this program will be effective in addressing risk factors that operate during pregnancy and childhood and compromise fetal and child development.

Trial registration: Current Controlled Trials ISRCTN16131117

Keywords: Randomized Controlled Trial, Child abuse, Social Class, Home nursing, Pregnancy

Background

Adverse events

The Adverse Childhood Experience Study concluded that living in poverty combined with other risk factors affects a person's physical and psychosocial health from childhood to adulthood. Moreover, even next generation(s) experience the same consequences as their parents suffered; they are all trapped in a vicious circle [1].

Low SES affects the child, even before birth. The unborn child is at risk of adverse pregnancy outcomes because of the negative health patterns of the parents. For example, women of low SES use substances during pregnancy more frequently than other women, and maternal substance use during pregnancy contributes to premature birth and low birth weight and is strongly associated with morbidity and mortality of the newborn as well as in childhood [2-6]. Having psychosocial problems during pregnancy leads to many complications, such as spontaneous abortion or preterm delivery; it also increases the chances that a child will later develop conduct problems [7-9]. The lack of structure

* Correspondence: crijnen@xs4all.nl
[3]Fier Fryslan, Child and Adolescent Trauma Center, Leeuwarden, The Netherlands
Full list of author information is available at the end of the article

BioMed Central

© 2011 Mejdoubi et al; licensee BioMed Central Ltd. This is an Open Access article distributed under the terms of the Creative Commons Attribution License (http://creativecommons.org/licenses/by/2.0), which permits unrestricted use, distribution, and reproduction in any medium, provided the original work is properly cited.

in the life of the children also increases their risk of displaying conduct problems and engaging in criminal activities. The low SES living area of the children is often unsafe; in this environment, children are more likely to experience injuries [10]. Child abuse is more common among these families. The consequences of child abuse are high: abused children have more psychosocial problems, low self esteem and morbidity [11]. In addition, abused children are more likely to engage in negative health behavior and criminal behavior when they get older [12]. Beyond these problems, the children are at risk of becoming a perpetrator of abuse themselves in the future [13-15].

When children living in low SES families grow up, they are more likely to have stress, anxiety and depression, because of their continued difficult lives. They usually have low income jobs and poor working conditions or are unemployed [16-20]. They live in bad housing conditions and struggle to live with limited financial resources. They can not pay for social activities, which leads to social isolation [21]. Their residential environment is also not favorable for their health and social network. They are more likely to engage in negative health related behavior, such as drinking alcohol, using drugs, smoking cigarettes and eating unhealthily [22]. In addition, chronic diseases like cardiovascular disease, diabetes and overweight are highest amongst these families. They therefore have a lower life expectancy and relatively more disease years [23-25].

Although several studies showed these consequences, only a few actually intervene on this issue. Meanwhile, the societal impact of the problems mentioned above is huge, not only in costs but also in higher use of resources and less participation in a positive society. Therefore, these problems should be effectively prevented wherever possible [26-31].

The Nurse Family Partnership

In the United States an intervention has been developed by David Olds that focuses on high-risk families, called the Nurse Family Partnership (NFP). Until now this is one of the few evidence-based interventions in the world for the prevention of disruptive disorder and child abuse. However, the effect of this program has not been studied yet outside the United States. David Olds and Alfons Crijnen reached agreement that the NFP-intervention could be adapted for use in the Netherlands under the condition that the effectiveness was examined in a trial. The Netherlands is thereby the first country outside the United States that was allowed to translate and culturally adapt the NFP into VoorZorg. Before the VoorZorg-intervention can be implemented on a larger scale in the existing and well organized Youth Health Care system in the Netherlands, it is important to study whether the VoorZorgprogram will be as effective as NFP in the United States compared to the usual care in the Netherlands.

VoorZorg in the Netherlands

To our knowledge there are no interventions in the Netherlands that start during pregnancy and are proven to be effective in reducing risk behavior among women and improving the health-outcomes of the child and mother. The available interventions that start after childbirth focus on the needs of the mother rather than placing the focus on the needs of the developing child by systematically addressing the risk factors for the child.

The main goal of VoorZorg is primary prevention of child abuse. Other goals are: to improve the outcomes of pregnancy by improving the mothers health during pregnancy (especially reduce their use of cigarettes and obtain prompt and reliable treatment for obstetric and other health problems such as depression), to improve the child's health and development by helping parents provide more competent care of their children, and to improve the mother's own personal development.

Theoretical Framework of VoorZorg

VoorZorg is based on three theories of human ecology:

Bandura's Self Efficacy Theory Bandura's model states that a person's behavior is determined by three factors: attitude, social influences and self-efficacy [Figure 1] [32,33]. The VoorZorg nurse is trained to affect a person's attitude towards behavioral change by providing the participant with knowledge about the negative effects of risk behavior. A person's intention to engage in a specific behavior is influenced by their social environment. The intervention focuses on the relationship of the participants with significant others, because they have a great influence on the participant. And VoorZorg focuses strongly on empowering the woman to stimulate her self efficacy [34,35].

Bronfenbrenner's ecological model Bronfenbrenner formulated a model to explain the influence of environment on the development of a person. An individual's immediate environment most strongly determines their development. According to Bronfenbrenner, mother-infant interaction is the most powerful predictor of the development of the person. If this interaction is strong and positive, the other environmental factors have less of an influence. When, for example, the child lives in a low SES area, but the mother-child interaction is strong and positive, SES has a less negative impact on the child's development. VoorZorg is used to instruct the mother on positive parenting skills and to empower the mother to have a positive influence on her child, despite the many environmental risk factors present [36].

Bowlby's Attachment theory Bowlby states that the quality of interaction between the caregiver and the child

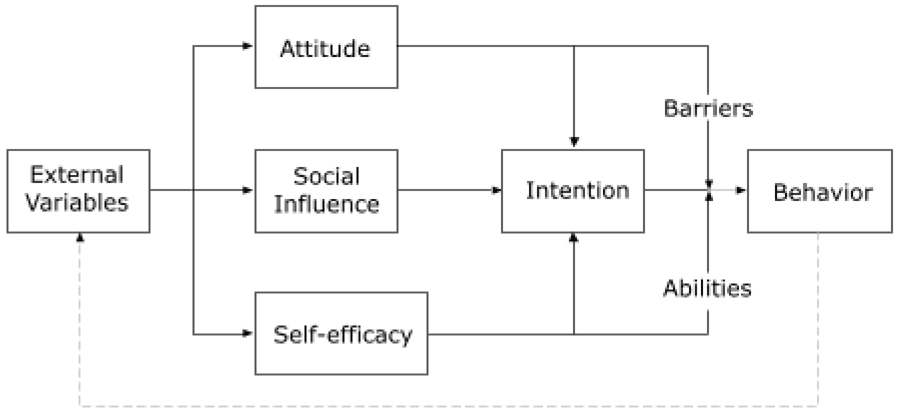

Figure 1 Bandura's self efficacy model, 1982.

is an important factor that determines the attachment of a child. In this theory, the quality of attachment in early life has a profound influence on the development of a child in later life. Disrupted attachment results in irreversible behavioral and psychosocial problems. Four types of attachment are described by Bowlby: 1) secure, 2) avoidant, 3) ambivalent/resistant and 4) disorganized. VoorZorg aims for a secure attachment between mother and child by discussing the importance of attachment during home visits, and by teaching the mothers parenting skills that are helpful in developing secure attachment [37,38].

Home visits

VoorZorg consists of approximately 10 home visits during pregnancy, 20 during the first life year of the child and 20 during the second life year. The visits are conducted by trained VoorZorg nurses. The visits are more frequent during the first month of the intervention and six weeks after birth, because these periods are important for the mother. The duration of each visit is between one hour and one and a half hour. The purpose of the visits is: structured behavioral changes, health education, discussing questions of the expectant mother, setting and maintaining realistic and achievable goals, increasing the mother's self-efficacy and involving the social network of the mother into the program. The VoorZorg nurses use three manuals that were designed for pregnancy, infancy and toddlerhood and focus on six domains: health status of the mother, child's health and safety, personal development of the mother, the mother as a role model, relation of the mother with her partner, family and friends and use of institutions. Each manual contains a full description of the visit. However, the visits are flexible and nurses are able to improvise when needed. It is important that VoorZorg nurses maintain a good relationship with the mother throughout the program.

Communication with stakeholders

Given the complexity of the tasks at hand, communication between the different stakeholders is considered very important to ensure successful implementation. To that end, several platforms were installed to discuss the implementation of VoorZorg, the monitoring and the study design: 1) a project group chaired by the initiator/child and adolescent psychiatrist consisting of experts from the EMGO+ Institute for Health and Care Research of the VU medical center, The Netherlands Youth Institute (NJi) and the Youth Health Care organization Evean; 2) an expert committee to settle arguments around inclusion; 3) a gathering of the managers of the ten participating Youth Health Care organizations; 4) a Committee of Advice consisting of experts at the national level. In addition to these platforms, prof. Olds and his co-workers are consulted by the researchers of VoorZorg.

The Care as Usual in the Netherlands

When pregnancy is confirmed (usually by a General Practioner) women visit a midwife. When complications are anticipated, the midwife refers the woman to an obstetrician. The aim of maternal health care is optimal pregnancy outcome. The caregiver (midwife or obstetrician) should offer health education, perform physical examinations and monitor the development of the fetus. Furthermore, the caregiver should support the parents and prepare them for the arrival of their baby. A pregnant woman will visit a midwife 4 times on average. After birth the mother can make use of the maternity care helper for a week. The costs of maternity care are reimbursed by health insurance companies. The maternity care helper visits the mother at home [39]. Her job is to take care of the mother, the newborn and the household and advice the mother about taking care for her baby and about breastfeeding the child.

In the Netherlands, every newborn will automatically be registered in a Youth Health Care organization (ambulatory well-baby clinic) to monitor the health and development of the child, and parents are supported in their parenthood. Furthermore, the child will be immunized

five times. Parents can also consult the Youth Health Care organization at any moment. This program is free of charge and voluntary, and the attendance rate is very high (95%) [40].

At week 1 (usually between 4 to 7 days after birth) and week 2 after birth, the parent will be visited at home. During the first visit the baby gets a heel prick by a trained nurse to test for several diseases. Early detection and treatment of those diseases is necessary to prevent serious mental and physical health problems. During this visit neonatal hearing screening is also conducted. The second visit will be performed by a Youth Health Care Nurse. During this visit, the child's health and environment will be observed and parents are informed about the development of their child. During four weeks after birth the parents can visit a Youth Health Care organization for a check-up. In total nine to eleven check-ups are performed until the child's second birthday. After the second birthday the consults will proceed in a less frequent schedule until the child's nineteenth birthday [41].

Objectives of this study

The implementation of VoorZorg in the Netherlands consists of three - partly overlapping - phases with their own objectives, preceded by preparation phase 0:

- Phase 1 aimed at translating, culturally adjusting and further developing the original intervention to accommodate the needs of pregnant women in the Netherlands and to address risk factors operating in the Dutch population. To identify women from the high-risk population, a screening procedure was developed and evaluated.
- Phase 2 aimed at assessing whether this intervention meets the needs of the at risk mothers and their yet-to-be-born children. Phase 2 also aimed at assessing whether the nurses visiting the mothers are capable of conducting the intervention as described in their protocols. This phase included an assessment of treatment integrity, and of the feasibility and adequacy of the intervention.
- Phase 3 aims at studying the effectiveness of VoorZorg in addressing the risk factors operating during pregnancy and early childhood that compromise fetal and early child development through a Randomized Controlled Trial.

Methods/design

PHASE 0

Preparation phase

This phase included the following elements:

- David Olds, founder of NFP, was contacted by Alfons Crijnen, a Dutch child physiatrist, and the two of them discussed the conditions of implementing NFP in the Netherlands. It was agreed that NFP needed to be adapted to the Dutch setting carefully to ensure implementation, and that the effectiveness should be examined through a Randomized Controlled Trial (RCT) prior to implementing NFP in the Netherlands on a wide scale;
- An overall plan including translation and adaptation, implementation, and assessment of effectiveness was developed;
- Stakeholders were invited to participate and a long-term commitment was requested;
- Grant proposals were written to collect financial resources;
- A project group was set up to translate and culturally adapt the NFP

PHASE 1

The Translation and cultural adaptation of the program

The translation and adaptation of the NFP for use in the Netherlands was conducted by the NJi together with the project group of VoorZorg and external experts.

The translation and adaption of the intervention occurred in steps:

1. Two members of the translation and development group and a manager of Youth Health Care organization Evean were trained in Denver (US) about the implementation of the NFP.
2. Program material was translated to Dutch. Furthermore, the material was adapted to fit in the Dutch Health Care System. In this way risk factors operating in the Dutch population were addressed and the needs of Dutch pregnant women were accommodated.
3. A reading group consisting of experts from the Netherlands Youth Institute (NJi) and the Youth Health Care organization Evean checked the translated and culturally adapted program material and made comments where necessary. The manuals were subsequently checked by representatives of the Dutch Societies for Midwives, Obstetricians and General Practitioners to ensure applicability in the Dutch health care system. Minor adjustments needed to be made. The adjusted parts of the manuals were then translated back into English by others in order to be verified by professor D. Olds.
4. A two-stage selection procedure was designed for recruitment of high-risk pregnant women.
5. The VoorZorg intervention was tested for applicability on a small-scale among eight high risk pregnant women.

PHASE 2

The Pilot implementation study

The pilot study was carried out in two Youth Health Care organizations in Zaanstreek-Waterland and Rotterdam. To conduct the evaluation, both qualitative data and quantitative data were collected from the 40 participating mothers who received the intervention and the VoorZorg nurses. The pilot study was evaluated by an independent research institute (The Trimbos institute).

The study showed, among other things, that the target population was reached adequately by means of the inclusion criteria formulated in VoorZorg. The program fulfilled the needs of the mothers and the mothers received significant support from the VoorZorg nurses. The VoorZorg nurses were able to carry out the intervention as described in the guidelines and the manuals were relevant to participants.

Training of the VoorZorg nurses

At the end of this phase twenty-five certified nurses were recruited by Youth Health Care organizations in twenty municipalities. The nurses were requested to comply with specified competences, including having a minimum of two years of working experience, affinity with high risk families, and experience with teaching parenting skills. All nurses received the following trainings to become a VoorZorg nurse: Video Home Training, training for pregnancy-, infant- and toddler period, and training about reducing smoking behavior with minimum intervention strategies (V-MIS). The trainers applied the training material that was used in the NFP.

In addition to these trainings, supervision at work on a weekly basis was a requirement for the execution of the program. The VoorZorg nurses were able to discuss difficulties in the implementation of the program with trained supervisors from their institution once a week. They could also discuss cases with other VoorZorg nurses during case conferences at the national level organized five times a year. The trainer or supervisor of the NJi could also be consulted. The maximum caseload for VoorZorg nurses with a full-time employment was 18 mothers.

PHASE 3

RCT

The third component of the program was the study on the effectiveness of VoorZorg through an RCT.

Design of the study

The study was designed as a double blind, parallel-group, randomized controlled trial (allocation ratio 1:1) starting before 28 weeks of pregnancy with a follow up of two and a half years. All data were handled confidentially. The Committee of Ethics on Human Research of the VU University medical center (Amsterdam, the Netherlands) approved the study design, protocols, information letters and informed consent form.

Study population

460 women were selected with a two-stage selection procedure from the year 2006 to 2009. The selection procedure is described more in detail elsewhere (Mejdoubi J., Heijkant van den S., Struijf E., Leerdam van F., Olds D., Crijnen A., Hirasing R., unpublished data). During the first stage professionals like General Practioners, midwifes, gynecologists and street corner workers recruited women in 20 different regions in the Netherlands based on the following criteria: Age below 26 years, low educational level (primary school or primary school and secondary school on a low level), pregnant with her first child, maximum 28 weeks of gestation, and understanding the Dutch language at a minimum level. During the second stage of the selection procedure the women were interviewed by VoorZorg nurses on several risk factors ((1) no or little social support, (2) a history of violence or abuse, (3) or still in a situation of domestic violence or neglect, (4) psychological problems, (5) financial problems, (6) unemployed or (7) housing problems, (8) alcohol problems, smoking or using drugs during pregnancy, or (9) having a non-realistic approach about motherhood) with the use of an inventory. Women who had at least one risk factor were included in the study. Furthermore, women had to understand the aim of the program and had to have the intention to complete the entire program. In addition, they were willing to participate in the study and be randomly assigned to an intervention or control group. Women who were found eligible for the study then signed, after the explanation of the study by the VoorZorg nurses, a written informed consent form. The participants were able to withdraw from the study at any time.

Outcome Measures

All participants' progress were measured six times during 16 to 28 and 32 weeks of pregnancy and during 2, 6, 12 and 24 months postpartum. The women received incentives for each measurement (a gift certificate of 15 euro's for each measurement and for the last measurement they received 30 euro's). All questionnaires were validated or were applied in other studies and published in the literature. Data about birth results were obtained from Youth Health Care organizations.

Interviewers

All measurements were performed by trained female interviewers who were blinded from randomization. The interviewers were recruited on strict competences; they were required to have a medicinal, nursing or pedagogic background. The interviewers were trained by a researcher of the VU University medical center according to the motivational training principles [42]. The interviewers were taught conversation skills to minimize social desirable answers and to increase reliability of the interviews. All interviewers met twice a year to discuss possible difficulties with each other. The researchers of the VU University

medical center were present during these meetings to advise them.

Primary outcome measures

Smoking cessation Specific questions were about numbers of cigarettes smoked at the gestational window of 16 to 28 weeks and during 32 weeks of pregnancy and 2 months postnatal. Smoking cessation by participant self report was measured at 32 weeks of pregnancy and 2 months postnatal.

Birth outcome Both nominal and ordinal birth weight were studied, for which four categories were made: very low <1000 g., low 1000-2500 g., normal 2500-4000 g. and high >4000 g. Gestational age was categorized in the following categories: extreme premature < 32 weeks, premature <37 weeks, normal gestation 37 to 41 weeks and serotine > 42 weeks. Dysmaturity was defined as a neonatal with a birth weight below the tenth percentile of the growth curve.

Domestic violence Women were asked at baseline detailed questions about whether they had experienced any violence in the past and in their current relationship. To measure whether participants had a history of abuse, the following questions were asked "*Have you ever been abused by your spouse or a significant other*?" Abuse was defined as being physically maltreated (being hit, punched, kicked, cut, burned) with or without a weapon and with or without injury. Sexual abuse was defined as forced sexual contact. To determine whether participants had been abused in the present relationship women were asked: "*Have you been hit, punched, kicked or in another way abused which resulted in physical injury, this year*?" If a woman answered these questions positively, detailed questions were asked about the perpetrator and frequency of the abuse. This questionnaire was translated from the NFP. Furthermore, the Revised Conflict Tactics Scale (CTS2) was conducted at 32 weeks of pregnancy and at the child's second birthday [43]. The Composite Abuse Scale (CAS) was conducted at 16 to 28 weeks of pregnancy [44].

Child development Child's development was measured at different periods:

At 6 months of age the Home Observation for Measurement of the Environment was conducted [45]. At 18 months, the language of the child and parental stress (Nijmeegse Ouderlijke Stress Index (NOSI)) were measured [46]. At the second birthday, the Child Behavior Checklist, Harsh Parenting and questions about raising the child were addressed [47].

Child abuse Data about prevalence of child abuse were obtained from a maltreatment reporting agency where both professionals and the general public can report cases (Advies & Meldpunt Kindermishandeling) [48]. A contact person from this agency was asked whether the child had been reported. In case of a reported child, further questions were asked about the perpetrator, the frequency, type and severity of the abuse. The contact person was also asked whether the report had been further investigated.

Secondary outcome measures

Number of risk factors for child abuse The number of risk factors present at baseline and two years after birth will be measured by self report by using the following questionnaires:

1. **Demographic factors** such as age, ethnicity, whether women received financial help or housing assistance from the government. Women were also asked whether the pregnancy was wanted. This questionnaire was translated from the registration forms used in the NFP.
2. **Depression** was measured by using the Edinburgh Post Natal Depression Scale (EPDS). The EPDS is effective in the detection of depression symptoms and can be used during pregnancy [49]. A total score higher than 13 indicates that the participant is suffering from depression and a score of 10 or higher indicates that the participant is possibly suffering from depression.
3. **Domestic violence** was addressed through detailed questions about violence in the past and the present by spouses or significant others.
4. **Substance use** was addressed by asking the women whether they smoked cigarettes, drank alcohol or used other drugs.

Sample size

Sample size calculation was based on finding effect in smoking cessation at the time of birth, 12 months and 24 months post partum. The numbers in the formula were based on findings from the effect study of the NFP [50]. In order to detect an average improvement of decreasing smoking by 4 cigarettes a day with a standard deviation of 8 cigarettes, a power of 80% and an alpha of 5% were used. This resulted in a sample size of 57. Given the fact that 25% of all women smoke at the start of the pregnancy, 228 participants in the control group and 228 participants in the intervention group should at least complete the pregnancy-component.

Randomization

A total of 460 women were included and randomized in strata by region and ethnicity into a control or intervention group by a researcher of the VU University medical center. Randomization was made blind by using a computer-generated list of random numbers (0, 1) in software SPSS 14.0 [51]. The researcher then informed the VoorZorg nurse about allocation. 237 women were assigned to the intervention group and were visited by trained VoorZorg nurses. 223 women were allocated to the control group and received the care as usual. A flow-chart of the RCT is shown in Figure 2. Women who lived in the same house as another participant of VoorZorg were not

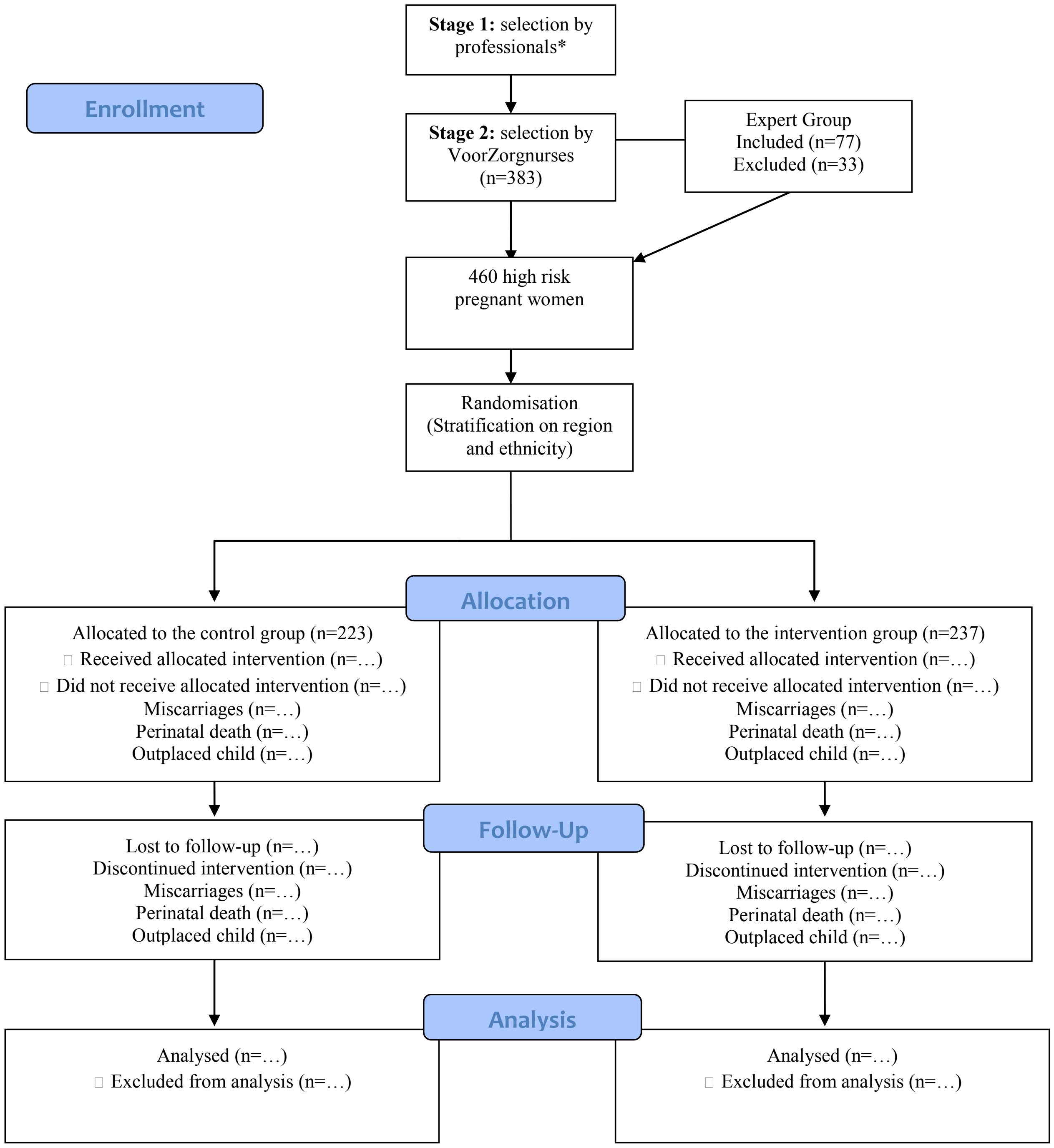

Figure 2 Flow of the participants through the study.* General Practioners, gynaecologists, midwifes, street corner workers etc.

randomized but assigned into the same treatment group to prevent contamination. This was relevant for one respondent who was assigned to the intervention group without randomization.

Analyses

The effectiveness of the VoorZorgprogram compared to care as usual will be analyzed with different statistical methods, using intention to treat analyses. Logistic Regression analyses will be used for comparing proportions between the intervention group and control group (smoking cessation, birth outcome, domestic violence and child abuse). Linear Regression analyses will be used for comparing means between the intervention and control group (numbers of cigarette smoked, birth

weight, gestational age, child development and number of risk factors for child abuse). Multilevel analyses will be used for analyzing longitudinal data. The multilevel and regression analyses using the (longitudinal) data as dependent variables were adjusted for possible confounders and were also checked for possible effect modification like age and ethnicity.

Discussion

This article presents the design of the Nurse Family Partnership (NFP) intervention in the Netherlands. The program material of the NFP has been translated and culturally adapted to fit in the Dutch health care system and is called VoorZorg. In addition, a two-stage selection procedure has been designed to include mothers-at-risk for abusing their child. The feasibility of the VoorZorg intervention was evaluated positively in a pilot study. At the moment an RCT is conducted to study the effectiveness of the VoorZorg program. It is hypothesized that with the VoorZorg program risk factors operating during pregnancy and early childhood that compromise fetal and early child development are addressed.

Strengths of this study include the random controlled design and the communication between different stakeholders and several experts (research and practice). An additional strength is that the program was first tested for feasibility in a pilot study. In this way the program could be improved in the last phase. Furthermore, the study results are generalisable for all high risk pregnant women, because this study is conducted in the practice setting and in different regions in the Netherlands and carried out in both urban and rural areas.

This study has some limitations as well. One is that each phase is financed by a different organization. This is not efficient because each project must be accounted for separately. Another limitation was that the effect study on VoorZorg was conducted by a University Medical Center in Rotterdam and during the study a different University Medical Center took over the project. However, the project manager of VoorZorg stayed involved in the study and continued to be a co-author. Also, some of the interviewers did not stay throughout the RCT. Participants were sometimes difficult to motivate to participate in the study, especially in the control group. Therefore, interviewers experienced several difficulties in making appointments for the measurements. It is for that reason that it is important to teach interviewers strong motivation skills.

In conclusion, this article presents the design of a program that aims at primary prevention of child abuse among high risk pregnant women. This program was initially implemented in the United States. Because in the Netherlands there is a notable lack of interventions that systematically address the risk factors during the prenatal and early infancy period, the NFP was adapted in the Netherlands. If the program proves to be effective in the Netherlands, it can be used by Youth Health Care organizations in preventing child abuse in high risk families.

Acknowledgements

We gratefully thank M. de Haan for collecting data, randomizing the participants and training the interviewers. We acknowledge K. Kooijman and G. Blokland for their contributions to the manual and training, and the VoorZorg nurses and their trainers, the interviewers and participants for their enthusiastic contribution. The first phases were funded by the Public Health Fund (Fonds OGZ) and the Netherlands Organisation for Health Research and Development (ZonMw) as well as by local communities. The RCT is funded by ZonMw, Academic Collaborative Centre, Child Health Care-North Holland-VU University medical center, participating Youth Health Care organizations and ZonMw Geestkracht for the research part and participating city councils for the implementation part.

Author details

[1]EMGO+ Institute for Health and Care Research, VU University Medical Center, Department of Public and Occupational Health, Amsterdam, The Netherlands. [2]GGD Hollands Noorden, Department of Youth Health Care, Schagen, The Netherlands. [3]Fier Fryslan, Child and Adolescent Trauma Center, Leeuwarden, The Netherlands.

Authors' contributions

AC had the original idea for the study, brought the stakeholders together, and organized the design of the study. ES, AC an FL were responsible for the translation and cultural adaption of the study and were involved in preparations for the study. AC was responsible for acquiring the grants for the first two phases; AC, RH and FL were responsible for acquiring the grant for the RCT. SH, FL, ES, AC and RH helped to coordinate the study and provided imput during the study. JM was responsible for the data collection, data analysis and writing the manuscript. All authors revised the manuscript for intellectual content. All authors read and approved the final version of the manuscript.

Competing interests

The authors declare that they have no competing interests.

Received: 12 October 2011 Accepted: 21 October 2011
Published: 21 October 2011

References

1. Felitti VJ, Anda RF, Nordenberg D, Williamson DF, Spitz AM, Edwards V, *et al*: **Relationship of childhood abuse and household dysfunction to many of the leading causes of death in adults - The adverse childhood experiences (ACE) study.** *American Journal of Preventive Medicine* 1998, **14**:245-258.
2. Nigg JT, Breslau N: **Prenatal smoking exposure, low birth weight, and disruptive behavior disorders.** *J Am Acad Child Adolesc Psychiatry* 2007, **46**:362-369.
3. Cnattingius S: **The epidemiology of smoking during pregnancy: smoking prevalence, maternal characteristics, and pregnancy outcomes.** *Nicotine Tob Res* 2004, **6(Suppl 2)**:S125-S140.
4. Barros FC, Bhutta ZA, Batra M, Hansen TN, Victora CG, Rubens CE: **Global report on preterm birth and stillbirth (3 of 7): evidence for effectiveness of interventions.** *BMC Pregnancy Childbirth* 2010, **10(Suppl1)**:S3.
5. Badshah S, Mason L, McKelvie K, Payne R, Lisboa PJG: **Risk factors for low birthweight in the public-hospitals at Peshawar, NWFP-Pakistan.** *Bmc Public Health* 2008, **8**.
6. Ventura SJ, Hamilton BE, Mathews TJ, Chandra A: **Trends and variations in smoking during pregnancy and low birth weight: Evidence from the birth certificate, 1990-2000.** *Pediatrics* 2003, **111**:1176-1180.
7. Mulder EJH, de Medina PGR, Huizink AC, Van den Bergh BRH, Buitelaar JK, Visser GHA: **Prenatal maternal stress: effects on pregnancy and the (unborn) child.** *Early Human Development* 2002, **70**:3-14.
8. Oberlander TF, Reebye P, Misri S, Papsdorf M, Kim J, Grunau RE: **Externalizing and attentional behaviors in children of depressed mothers treated with a selective serotonin reuptake inhibitor**

antidepressant during pregnancy. *Archives of Pediatrics & Adolescent Medicine* 2007, **161**:22-29.
9. O'Connor TG, Heron J, Golding J, Beveridge M, Glover V: **Maternal antenatal anxiety and children's behavioural/emotional problems at 4 years - Report from the Avon Longitudinal Study of Parents and Children.** *British Journal of Psychiatry* 2002, **180**:502-508.
10. Aneshensel CS, Sucoff CA: **The neighborhood context of adolescent mental health.** *Journal of Health and Social Behavior* 1996, **37**:293-310.
11. Durkin MS, Davidson LL, Kuhn L, Oconnor P, Barlow B: **Low-Income Neighborhoods and the Risk of Severe Pediatric Injury - A Small-Area Analysis in Northern Manhattan.** *American Journal of Public Health* 1994, **84**:587-592.
12. Afifi TO, Enns MW, Cox BJ, de GR, ten HM, Sareen J: **Child abuse and health-related quality of life in adulthood.** *J Nerv Ment Dis* 2007, **195**:797-804.
13. Thornberry TP, Henry KL, Ireland TO, Smith CA: **The Causal Impact of Childhood-Limited Maltreatment and Adolescent Maltreatment on Early Adult Adjustment.** *Journal of Adolescent Health* 2010, **46**:359-365.
14. Christian CW, Sege RD: **Policy Statement-Child Fatality Review.** *Pediatrics* 2010, **126**:592-596.
15. Felitti VJ, Anda RF, Nordenberg D, Williamson DF, Spitz AM, Edwards V, *et al*: **Relationship of childhood abuse and household dysfunction to many of the leading causes of death in adults - The adverse childhood experiences (ACE) study.** *American Journal of Preventive Medicine* 1998, **14**:245-258.
16. Everson SA, Maty SC, Lynch JW, Kaplan GA: **Epidemiologic evidence for the relation between socioeconomic status and depression, obesity, and diabetes.** *Journal of Psychosomatic Research* 2002, **53**:891-895.
17. Felitti VJ, Anda RF, Nordenberg D, Williamson DF, Spitz AM, Edwards V, *et al*: **Relationship of childhood abuse and household dysfunction to many of the leading causes of death in adults - The adverse childhood experiences (ACE) study.** *American Journal of Preventive Medicine* 1998, **14**:245-258.
18. Cohen A, Gilman SE, Houck PR, Szanto K, Reynolds CF: **Socioeconomic status and anxiety as predictors of antidepressant treatment response and suicidal ideation in older adults.** *Social Psychiatry and Psychiatric Epidemiology* 2009, **44**:272-277.
19. Taylor R, Page A, Morrell S, Harrison J, Carter G: **Mental health and socio-economic variations in Australian suicide.** *Social Science & Medicine* 2005, **61**:1551-1559.
20. Miech RA, Caspi A, Moffitt TE, Wright BRE, Silva PA: **Low socioeconomic status and mental disorders: A longitudinal study of selection and causation during young adulthood.** *American Journal of Sociology* 1999, **104**:1096-1131.
21. Bartley M, Popay J, Plewis I: **Domestic Conditions, Paid Employment and Womens Experience of Ill-Health.** *Sociology of Health & Illness* 1992, **14**:313-343.
22. Aneshensel CS, Sucoff CA: **The neighborhood context of adolescent mental health.** *Journal of Health and Social Behavior* 1996, **37**:293-310.
23. Janssen I, Boyce WF, Simpson K, Pickett W: **Influence of individual- and area-level measures of socioeconomic status on obesity, unhealthy eating, and physical inactivity in Canadian adolescents.** *American Journal of Clinical Nutrition* 2006, **83**:139-145.
24. Carbonneau R, Tremblay RE, Vitaro F, Dobkin PL, Saucier JF, Pihl RO: **Paternal alcoholism, paternal absence and the development of problem behaviors in boys from age six to twelve years.** *Journal of Studies on Alcohol* 1998, **59**:387-398.
25. Bartley M, Popay J, Plewis I: **Domestic Conditions, Paid Employment and Womens Experience of Ill-Health.** *Sociology of Health & Illness* 1992, **14**:313-343.
26. Everson SA, Maty SC, Lynch JW, Kaplan GA: **Epidemiologic evidence for the relation between socioeconomic status and depression, obesity, and diabetes.** *Journal of Psychosomatic Research* 2002, **53**:891-895.
27. Bartley M, Popay J, Plewis I: **Domestic Conditions, Paid Employment and Womens Experience of Ill-Health.** *Sociology of Health & Illness* 1992, **14**:313-343.
28. Louwman WJ, Aarts MJ, Houterman S, van Lenthe FJ, Coebergh JWW, Janssen-Heijnen MLG: **A 50% higher prevalence of life-shortening chronic conditions among cancer patients with low socioeconomic status.** *British Journal of Cancer* 2010, **103**:1742-1748.
29. Mulia N, Ye Y, Zemore SE, Greenfield TK: **Social Disadvantage, Stress, and Alcohol Use Among Black, Hispanic, and White Americans: Findings From the 2005 US National Alcohol Survey.** *Journal of Studies on Alcohol and Drugs* 2008, **69**:824-833.
30. Rask K, O'Malley E, Druss B: **Impact of socioeconomic, behavioral and clinical risk factors on mortality.** *Journal of Public Health* 2009, **31**:231-238.
31. Clark AM, DesMeules M, Luo W, Duncan AS, Wielgosz A: **Socioeconomic status and cardiovascular disease: risks and implications for care.** *Nature Reviews Cardiology* 2009, **6**:712-722.
32. Corso PS, Fertig AR: **The economic impact of child maltreatment in the United States: are the estimates credible?** *Child Abuse Negl* 2010, **34**:296-304.
33. Belsky J, Bell B, Bradley RH, Stallard N, Stewart-Brown SL: **Socioeconomic risk, parenting during the preschool years and child health age 6 years.** *European Journal of Public Health* 2007, **17**:508-513.
34. Bandura A: **Self-Efficacy - Toward A Unifying Theory of Behavioral Change.** *Psychological Review* 1977, **84**:191-215.
35. Bandura A: **Self-Efficacy Mechanism in Human Agency.** *American Psychologist* 1982, **37**:122-147.
36. Bronfenbrenner U: **Ecology of the Family As A Context for Human-Development - Research Perspectives.** *Developmental Psychology* 1986, **22**:723-742.
37. Bowlby J: **Attachment theory and its therapeutic implications.** *Adolesc Psychiatry* 1978, **6**:5-33.
38. Zegers MA, Schuengel C, Van IJzendoorn MH, Janssens JM: **Attachment and problem behavior of adolescents during residential treatment.** *Attach Hum Dev* 2008, **10**:91-103.
39. Basis prenatale zorg: **Omschrijving van het probleem.**, 18-4-2002. NVOG. Ref Type: Report.
40. Verbrugge HP: **Youth health care in The Netherlands: a bird's eye view.** *Pediatrics* 1990, **86**:1044-1047.
41. Hirasing RA, Ivan Zaal MAE, Meulmeester JF, Verbrugge JF: *Child health in the Netherlands* Leiden, The Netherlands; 1997.
42. Lane C, Hood K, Rollnick S: **Teaching motivational interviewing: using role play is as effective as using simulated patients.** *Medical Education* 2008, **42**:637-644.
43. Straus MA, Hamby SL, BoneyMcCoy S, Sugarman DB: **The revised Conflict Tactics Scales (CTS2) - Development and preliminary psychometric data.** *Journal of Family Issues* 1996, **17**:283-316.
44. Hegarty K, Sheehan M, Schonfeld C: **A multidimensional definition of partner abuse: Development and preliminary validation of the composite abuse scale.** *Journal of Family Violence* 1999, **14**:399-415.
45. Bradley RH, Caldwell BM: **Home Observation for Measurement of the Environment - Revision of the Preschool Scale.** *American Journal of Mental Deficiency* 1979, **84**:235-244.
46. Brock dAJ, Vermulst AA, Gerris JRM: **De Nijmeegse Ouderlijke Stress Index.** *Tijdschrift voor primaire leefvormen* 1990, **2**:57-75.
47. Achenbach TM, Dumenci L: **Advances in empirically based assessment: Revised cross-informant syndromes and new DSM-oriented scales for the CBCL, YSR, and TRF: Comment on Lengua, Sadowksi, Friedrich, and Fisher (2001).** *Journal of Consulting and Clinical Psychology* 2001, **69**:699-702.
48. Bouwmeester-Landweer M: *Early home visitation in families at risk for child maltreatment* 2006.
49. Cox JL, Holden JM, Sagovsky R: **Detection of Postnatal Depression - Development of the 10-Item Edinburgh Postnatal Depression Scale.** *British Journal of Psychiatry* 1987, **150**:782-786.
50. Olds DL, Henderson CR Jr, Tatelbaum R, Chamberlin R: **Improving the delivery of prenatal care and outcomes of pregnancy: a randomized trial of nurse home visitation.** *Pediatrics* 1986, **77**:16-28.
51. SPSS Incorporation: **SPSS 14.0 for Windows. 2005.** Chicago;, Ref Type: Computer Program.

Pre-publication history
The pre-publication history for this paper can be accessed here:
http://www.biomedcentral.com/1471-2458/11/823/prepub

doi:10.1186/1471-2458-11-823
Cite this article as: Mejdoubi *et al.*: **Addressing risk factors for child abuse among high risk pregnant women: design of a randomised controlled trial of the nurse family partnership in Dutch preventive health care.** *BMC Public Health* 2011 **11**:823.
